# Towards automated behaviour monitoring in wildlife: a review of machine learning approaches using accelerometer data

**DOI:** 10.7717/peerj.21069

**Published:** 2026-05-11

**Authors:** Lorène Jeantet, W. Chris Oosthuizen, Damien Chevallier, Emmanuel Dufourq

**Affiliations:** 1 African Institute for Mathematical Sciences, Muizenberg, Western Cape, South Africa; 2 African Institute for Mathematical Sciences Research and Innovation Centre, Kigali, Rwanda; 3 Department of Mathematical Sciences, Stellenbosch University, Stellenbosch, South Africa; 4 Marine Apex Predator Research Unit, Department of Zoology and Institute for Coastal and Marine Research, Nelson Mandela University, Gqeberha, South Africa; 5 Unité de Recherche BOREA, MNHN, CNRS 8067, SU, IRD 207, UA, Station de Recherche Marine de Martinique, Centre National de la Recherche Scientifique, Les Anses d’Arlet, Martinique; 6 Computer Science, University of Northern Iowa, Cedar Falls, Iowa, United States

**Keywords:** Bio-logging, Deep learning, Machine learning, Accelerometer, Animal behaviour classification, Wildlife monitoring

## Abstract

Monitoring animal behaviour provides critical insights into species ecology and offers essential information for guiding management and conservation efforts. Among the various approaches used to study behaviour, bio-logging—the use of animal-borne data recorders—has emerged as a valuable tool for observing animals in their natural habitats while minimising human disturbance. A major advancement in bio-logging has been the integration of accelerometers, which enable high-resolution analysis of movement and activity in free-ranging animals. By identifying movement and postural patterns captured by accelerometers, it becomes possible to associate specific accelerometric sequences with distinct behaviours. This task—automatically classifying repetitive accelerometric patterns linked to behaviours—can be achieved using machine-learning algorithms. The first ecological study applying machine learning to identify animal behaviours from accelerometer data was published in 2009. Since then, numerous studies have expanded this approach across a wide range of species, employing diverse methodological frameworks that make it challenging for new practitioners to identify best practices. The aim of this review is to provide a comprehensive overview of accelerometer-based behavioural identification in ecology using machine learning. We summarise the range of species and applications investigated, highlight key methodological trends, and offer practical guidance for researchers seeking to apply this approach. Based on 125 studies, we show that current practices largely rely on a general framework combined with species-specific adaptations. This has led to a predominance of species-focused methodological studies and limited generalisation across taxa. More importantly, the high diversity of datasets, combined with highly variable validation procedures—sometimes insufficient to assess model generalisation to novel data—prevents robust comparisons, limiting the identification of broadly applicable approaches. We therefore seek to re-establish a clear methodological framework that enables meaningful cross-study comparisons. Although methodological advances have been relatively modest until recently, 2024 marks a turning point, with a growing number of studies applying deep learning approaches that hold promise for improving model generalisation. While deep learning remains less widely adopted in ecology than in human or livestock behaviour recognition, leveraging advances from these fields and fostering interdisciplinary collaboration will be essential to accelerate progress. In particular, developments in real-time monitoring offer strong potential to enhance conservation efforts, an important next step in bio-logging. Nevertheless, despite increasing automation and generalisation, reliable behavioural classification models will continue to depend on robust ground-truth data and strong expertise in the natural history and biology of the study organisms.

## Introduction

Animal behaviour is a fundamental driver of ecological processes, shaping species distributions, population dynamics, and ecosystem functioning (Mortelliti, 2023). From foraging decisions to social interactions, behavioural responses to internal and external stimuli mediate how individuals interact with conspecifics, other species, and their environment. Animals often display behavioural shifts as an early response to environmental change, so understanding these responses—whether to natural or human-induced disturbances—can help improve conservation management (Greggor et al., 2016; Amorim, 2025). Studying wild animals in their natural environments is an integral part of behavioural ecology, as it allows researchers to link behaviour with the ecological interactions and environmental pressures that shape it. Yet, direct behavioural observation in free-ranging populations is often difficult. The presence of human observers can alter the behaviour of the species being studied (Schneirla, 1950; Jack et al., 2008), and many species inhabit environments where direct observation is impractical or impossible. These challenges frequently render remote monitoring techniques essential for investigating animal behaviour under natural conditions.

**Figure 1:**
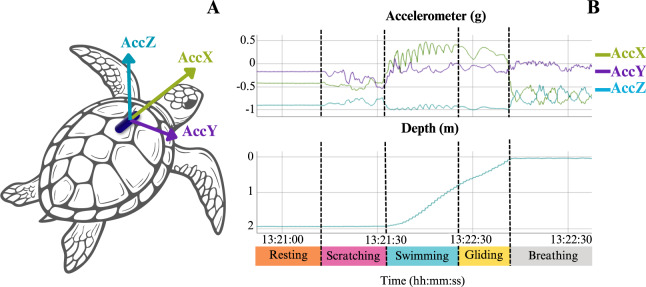
Illustration of a tri-axial accelerometer deployed on a sea turtle, with an example of raw acceleration and depth data associated with observed behaviours. (A) Illustration showing a sea turtle equipped with a tri-axial accelerometer, indicating the surge (AccX), sway (AccY), and heave (AccZ) axes. (B) Example of raw acceleration and depth data recorded from a green turtle (*Chelonia mydas*) in Martinique, linked to observed behaviours (adapted from Jeantet et al. (2020)).

Bio-logging, which involves attaching data recorders directly to animals, has revolutionised the study of animal behaviour (Brown et al., 2013; Wilmers et al., 2015; Fehlmann & King, 2016). Bio-logging enables data collection even when animals move rapidly over large distances, navigate harsh weather conditions, dive underwater, or remain active at night (Kooyman, 2004; Ropert-Coudert & Wilson, 2005). In recent decades, bio-logging evolved from simple location tracking tags to multi-sensor systems that provide deeper insights into animal behaviour and open new research domains (Wilmers et al., 2015; Watanabe & Papastamatiou, 2023). One major advancement in bio-logging is the integration of accelerometers, allowing high-resolution analysis of movement and activity in free-ranging animals. In the field of behavioural ecology, an accelerometer operates as a piezoelectric spring sensor, generating a voltage signal when subjected to deformation. This deformation is influenced by both static acceleration, associated with gravitational force and body posture, and inertial acceleration, originating from dynamic movement, with the resulting wave-like signal being proportional to the acceleration (Shepard et al., 2008a; Brown et al., 2013). Typically, an accelerometer consists of three sensors arranged orthogonally to represent posture and movements in three dimensions (Fig. 1A). By identifying the movement and postural patterns captured by the accelerometer, it becomes possible to link accelerometric sequences to specific behaviours (Fig. 1B), a powerful tool for remotely studying wildlife behaviour.

In wildlife applications, accelerometers often sample at high frequencies (*e.g*., up to 200 Hz; Holland et al., 2009; Brown et al., 2013) to capture the fine-scale movements of animals. When deployed continuously over several days, these high sampling rates generate very large datasets containing millions of data points, which can be challenging to process and analyse. For instance, a 10-day deployment with multiple sensors (tri-axial accelerometer, tri-axial gyroscope, and depth sensor) recording at 20 Hz can produce a data file exceeding 3 GB. While this technological advance offers unprecedented insights into animal behaviour, it also introduces new challenges, particularly in efficiently managing large datasets and developing analytical methods to transform raw sensor signals into meaningful behavioural information.

Although some behaviours—such as the rapid movements associated with prey capture in marine predators—can be identified through simple threshold-based analyses (Yoda et al., 2001; Kokubun et al., 2011; Watanabe & Takahashi, 2013), more sophisticated approaches are required to identify distinct and repeatable signal patterns, and to reliably link these patterns to specific behaviours. Consequently, the automation of data processing has emerged as a critical challenge in accelerometry, highlighting the need for methods that can automatically detect these characteristic signal patterns. Two primary categories of automated analysis approaches are commonly distinguished: unsupervised learning algorithms and supervised learning algorithms (Box 1). Unsupervised learning algorithms identify common features across unlabelled accelerometer data and group these into clusters. Subsequently, the analyst can assign distinct behaviour classes to each identified cluster. In unsupervised learning, behaviours are therefore inferred or linked to clusters without using behavioural observations as the training input (Wang, 2019). In contrast, supervised learning algorithms use behavioural observation as labels (*i.e*., labelled accelerometer data) to train the algorithm to identify the discriminating features of accelerometer sequences. To date, many of the machine learning methods used for behaviour identification have been based on decision tree algorithms, such as classification and regression tree (later referred to simply as Decision Tree, DT), Random Forest (RF), and Gradient Boosting (GB), or on the linear hyperplane separation principle underlying Support Vector Machines (SVM) (Nathan et al., 2012; Campbell et al., 2013; see Box 2 for definitions). Deep learning, which has enabled major methodological advances such as automated feature extraction, improved pattern recognition, and the analysis of high-dimensional data, achieving state-of-the-art performance across a wide range of tasks (LeCun, Bengio & Hinton, 2015; van Dis et al., 2023; Barbadilla-Martínez et al., 2025), may also reshape accelerometer analysis in the future. Computer-vision based deep learning methods are widely applied in ecology (*e.g*., in bioacoustics and analysis of image or video data, Stowell, 2022; Tuia et al., 2022; Chimienti et al., 2025). By contrast, the adoption of deep learning in accelerometry within the field of ecology is still limited, but extensive applications exist in other related fields, such as Human Activity Recognition (HAR, Wang et al., 2019; Zhang et al., 2022; Kumar, Chauhan & Awasthi, 2023) and livestock welfare and productivity monitoring (Balasso et al., 2023; Mao et al., 2023; Turner et al., 2023). Therefore, in a rapidly evolving scientific landscape influenced by machine learning, it is crucial to critically assess the methodologies used in accelerometer-based behavioural identification for wildlife monitoring, particularly in light of the potential of deep learning methods.

Box 1 **Definition of the main terms of artificial intelligence**

**Table:** 

**Artificial Intelligence (AI):** A field of computer science that develops techniques and algorithms enabling machines to perform tasks associated with human intelligence, such as reasoning, problem-solving, and decision-making.	
**Machine Learning:** A subfield of AI that uses data-driven algorithms to allow systems to automatically learn patterns and improve performance on specific tasks without being explicitly programmed. Machine learning is broadly categorised into two types: supervised learning and unsupervised learning.	
**Deep Learning:** A specialised branch of machine learning that enables high levels of abstraction through a series of linear and nonlinear mathematical operations organised within a neural network.	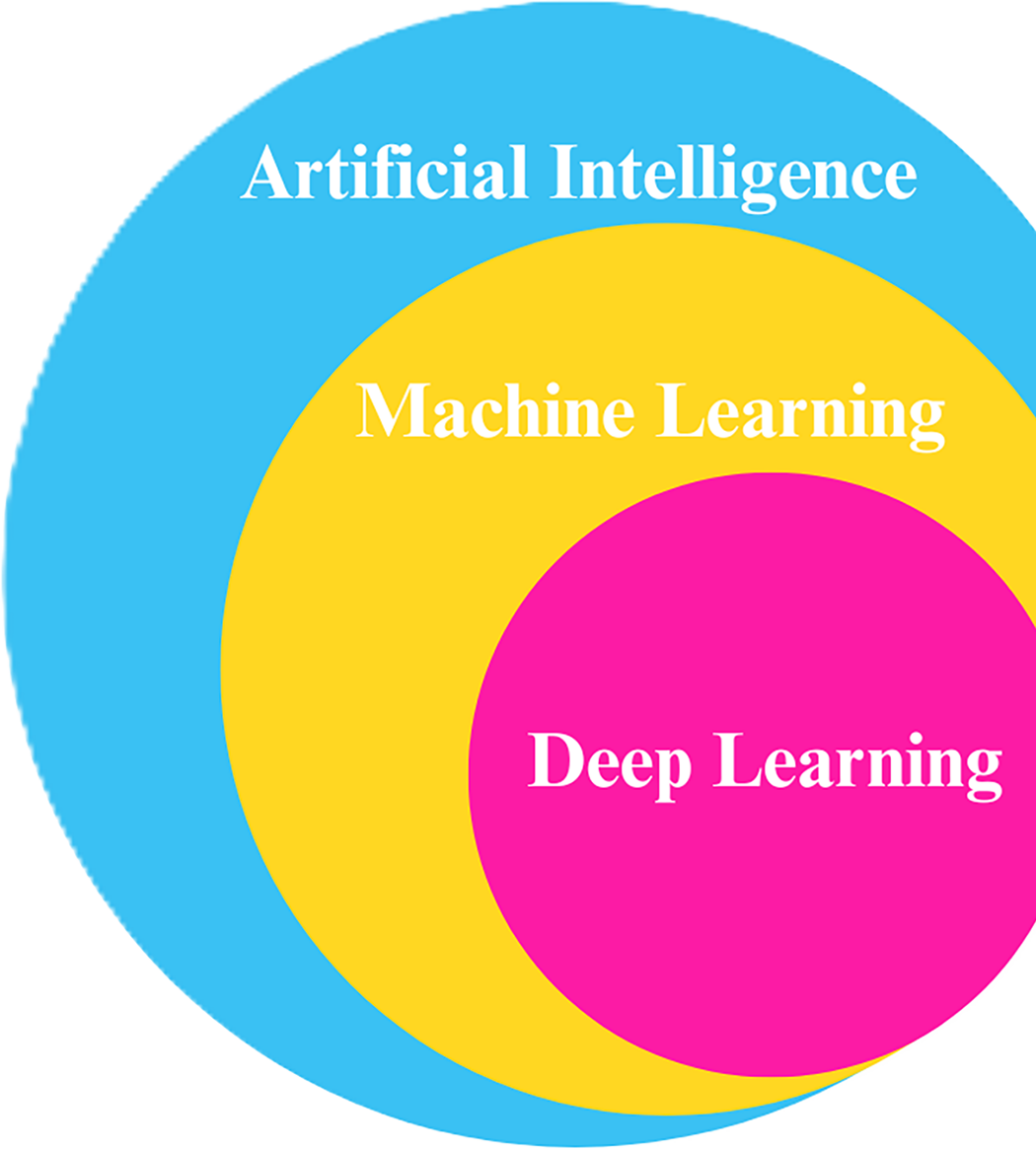
**Supervised Learning:** A machine learning approach in which an algorithm is trained on labelled data, learning to map inputs to the outputs by generalising from known examples.	
**Unsupervised Learning:** A machine learning approach used with unlabelled data, where the algorithm identifies hidden patterns, relationships, or structures within the data without predefined outputs.

Box 2 **Definition of the main supervised machine learning algorithms used in acceleration-based behaviour identification in ecology**

**Decision Tree (DT):** A classification algorithm that applies a sequence of hierarchical rules to predict behaviours. It builds a binary tree in which each internal node represents a threshold-based decision on a descriptive feature, recursively splitting the dataset into two subsets. This process continues until a predefined stopping criterion is reached. The predicted behaviour is then determined by following the path of decisions along the tree from the root to a terminal (leaf) node.

**Random Forest (RF):** An ensemble learning algorithm that combines the predictions of multiple DTs to improve classification performance. Each tree is trained on a different bootstrap sample of the dataset, and at each node, a random subset of features is considered to determine the best split. The final prediction is obtained by aggregating the outputs of all individual trees, typically through majority voting for classification tasks.

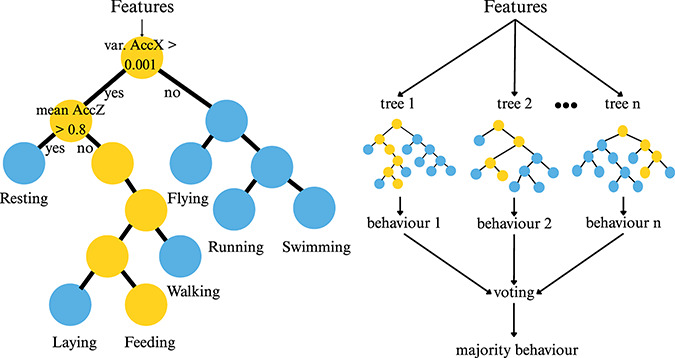

**Gradient Boosting (GB):** An ensemble learning algorithm that builds a predictive model by sequentially adding weak learners, typically DTs. Each new tree is trained to correct the residual errors made by the ensemble of previously combined trees. This is achieved by minimising a loss function using gradient descent, allowing the model to focus on the most difficult-to-predict observations.

**Support Vector Machine (SVM):** A classification algorithm that constructs an optimal hyperplane to separate data points from different behaviours. This hyperplane is chosen to maximise the distance between itself and the nearest data points from each behaviour. While SVM is inherently designed for binary classification, multi-class problems can be addressed by combining multiple binary classifiers using strategies such as one-vs-all or one-vs-one.

**K-Nearest Neighbours (K-NN):** An algorithm that assigns a new data point to the majority behaviour of its *k* closest neighbours in the feature space, as determined by a chosen distance metric. K-NN belongs to the family of ‘lazy learning’ models, meaning that it does not involve an explicit training phase but instead stores the entire training dataset.

**Artificial Neural Network (ANN):** A computational model inspired by biological neural networks, composed of interconnected nodes or ‘neurons’ organised in layers that process information by summing their inputs with weights and applying an activation function, often non-linear, to generate an output. The most commonly used ANN in this literature review consists of a single hidden layer and is therefore not considered deep learning. In contrast, ANNs with multiple hidden layers fall under the category of deep learning.

**Deep learning:** A model based on ANN with multiple processing layers to progressively extract higher-level features from data. The term “deep” refers to the presence of multiple layers, ranging from a few to hundreds, stacked within the network. Deep learning encompasses a wide variety of architectures, each defined by the specific organisation and interaction of layers and operations tailored to the task at hand.

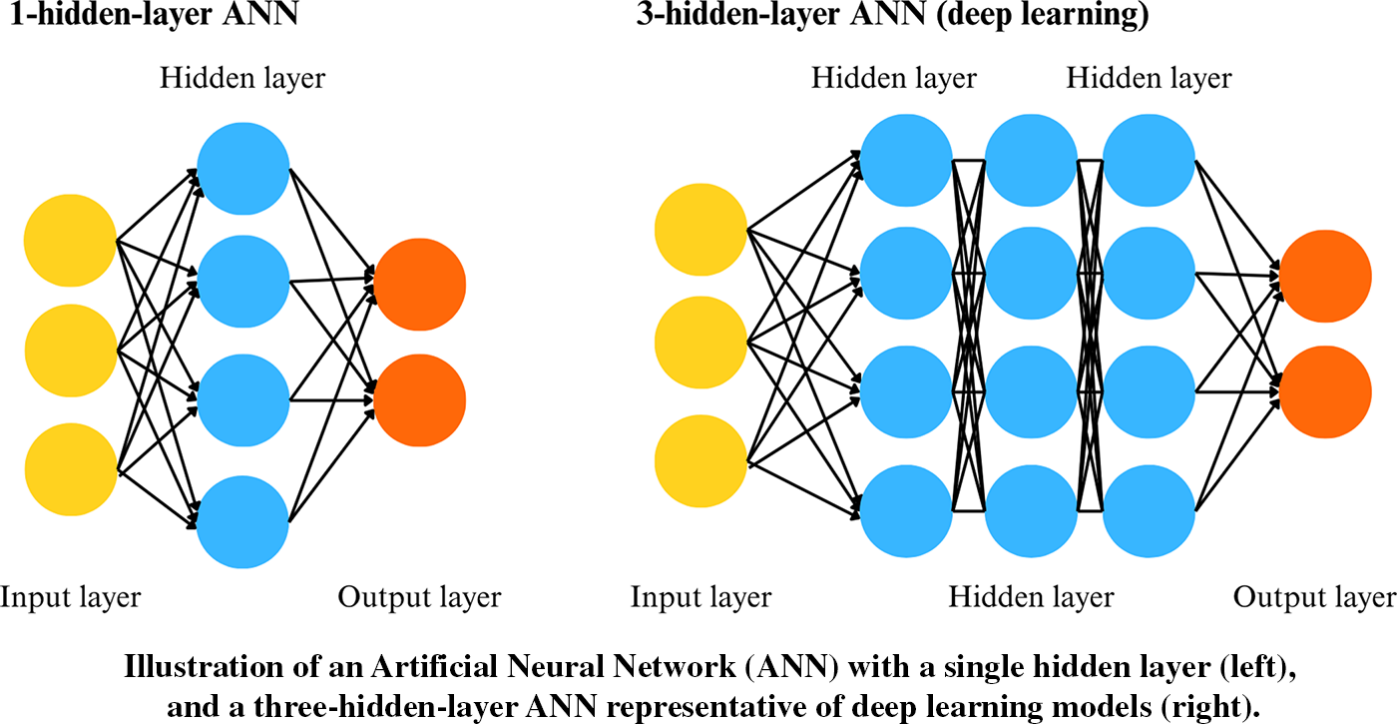

**Discriminant Analysis (DA):** A classification method that models the relationship between input features and categorical outcomes by assuming that each class follows a multivariate normal distribution with class-specific parameters. The algorithm estimates the probability that a given observation belongs to each class and assigns it to the one with the highest posterior probability. Linear Discriminant Analysis (LDA) assumes equal covariance matrices across classes, resulting in linear decision boundaries, while Quadratic Discriminant Analysis (QDA) allows for class-specific covariances, leading to more flexible, non-linear boundaries.

**Logistic Regression (LR):** A linear classification model that analyses the relationship between predictor variables (features) and a categorical output variable. It estimates the probability that a given observation belongs to a particular class based on the input features. Unlike linear regression, which predicts continuous outcomes, LR uses the logistic (sigmoid) function to map predicted values to probabilities between 0 and 1. Observations are then assigned to the class with the highest predicted probability.

This study presents a comprehensive review of machine learning methods used in ecology to automatically identify wild animal behaviour from accelerometer data. Our focus is on studies employing supervised or unsupervised algorithms, emphasising both methodological development and practical applications. While similar reviews exist for humans and domesticated animal research fields (Narayanan et al., 2020; Riaboff et al., 2022; Kumar, Chauhan & Awasthi, 2023; Tran et al., 2024), no systematic review has yet specifically focused on wild animals. One exception is a recent review by Wilson et al. (2025), which focuses specifically on the validation of supervised machine learning algorithms in accelerometer-based animal behaviour classification. To address this gap, we structure our review around three main sections, each further divided into subsections that explore key aspects of this emerging field. “Section 1: Analysis of the reviewed studies” aims to highlight the value of automatic behavioural identification and its current applications in wildlife monitoring. Specifically, we examine which species have been studied using machine learning and accelerometer data, and the objectives of those studies. “Section 2: Methods used to automatically classify behaviour from accelerometer data” reviews the methodologies used to train machine learning models for automatic behaviour classification, with the aim of identifying common practices and offering practical guidance for researchers seeking to adopt this approach. Given the difficulty of addressing each methodological step—from bio-logger deployment through to behaviour identification—this review narrows its scope to the data analysis component. Pre-deployment considerations, such as the choice of sensors beyond accelerometers, sample size requirements, sampling frequencies and logger placement, are not discussed in this review. However, this information was documented for each study and is provided in the Supplemental Materials, together with the full list of reviewed papers. Instead, “Section 2: Methods used to automatically classify behaviour from accelerometer data” focuses on signal validation for supervised learning, data preprocessing, and model selection. Finally, “Section 3: Identification of gaps and future directions for automatic behavioural identification from accelerometer data using machine learning” identifies remaining gaps and outlines future directions for monitoring wild animal behaviour with accelerometer data and machine learning.

## Materials and Methods

This literature review was conducted through keyword-based searches on Web of Science. Our specific query was designed to identify studies that develop or apply machine learning methods to accelerometer data capturing animal movement:

(acceler* OR sensor OR biologger OR bio-logger OR gyroscope) AND (“machine learning” OR “supervised learning” OR “unsupervised learning” OR “deep learning” OR “automatic identification” OR “neural network”) AND (animal OR bird OR cetacean OR mammal) AND behavio*

While this search string does not capture every accelerometer application, it specifically targets studies focused on machine learning approaches for behavioural identification. We believe this query captured a representative sample of relevant literature, even if some individual studies were missed. Initially, we compiled every study that employed accelerometer data to automatically classify animal behaviours—covering livestock, poultry, companion animals, and wildlife—to capture the full breadth of existing research. Subsequently, articles focusing specifically on livestock (including poultry and companion animals) were separated, and only those pertaining to wildlife monitoring were retained for further analysis. Lists of both the livestock and wildlife studies are provided in the Supplemental Materials.

The literature search described above yielded 381 articles on the 10^th^ of November 2023. We excluded off-topic articles, studies that did not use machine learning or accelerometer methods, and review articles or articles that were not peer-reviewed. This reduced the total number of articles to 213, with 62 articles directly relevant to wildlife monitoring. To augment the literature retrieved in the initial search, a second search was conducted using Google Scholar, with the following query: accelerometer bio-logging machine learning animal behaviour-human. This search, conducted on the 18th of November 2023, yielded 233 articles. Twenty-seven additional articles on wildlife monitoring that applied machine learning to accelerometer data were identified and added to the database.

Since November 2023, additional searches were conducted on Web of Science and Google Scholar using the same methodology to retrieve the most recent publications (*n* = 24). The last search, completed on 25 July 2025, included all articles published up to 31 December 2024. We were able to augment this structured literature search with 12 additional articles, obtained through scientific networks and personal databases. Consequently, a total of 125 articles on the automatic identification of wildlife behaviour using accelerometers and machine learning were included in this review. The complete list of articles, along with the information extracted from each study, is provided in the Supplemental Materials.

## Results

### Section 1: Analysis of the reviewed studies

#### Temporal trends

**Figure 2:**
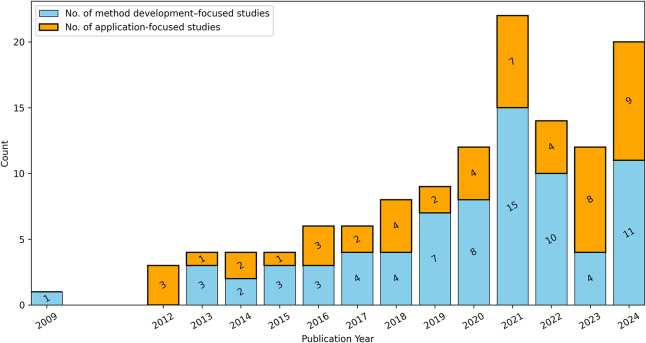
Annual number of publications on the automatic identification of wildlife behaviour using accelerometers and machine learning. Blue bars represent methodological studies focused on developing or adapting algorithms using data from wild or captive animals, while orange bars indicate applications to unlabelled datasets from wild populations.

Since the 1990s, accelerometers have been used as bio-logging sensors to study wild animal behaviour (Scheibe et al., 1998; Sellers, Varley & Waters, 1998; Yoda et al., 1999), with their use expanding significantly since the late 2000s (Brown et al., 2013). Machine learning was first used in 2009 to identify wildlife behaviour from accelerometer data, and since then, the number of studies applying these methods to accelerometer data has steadily increased (Fig. 2).

#### Taxonomic diversity

**Figure 3:**
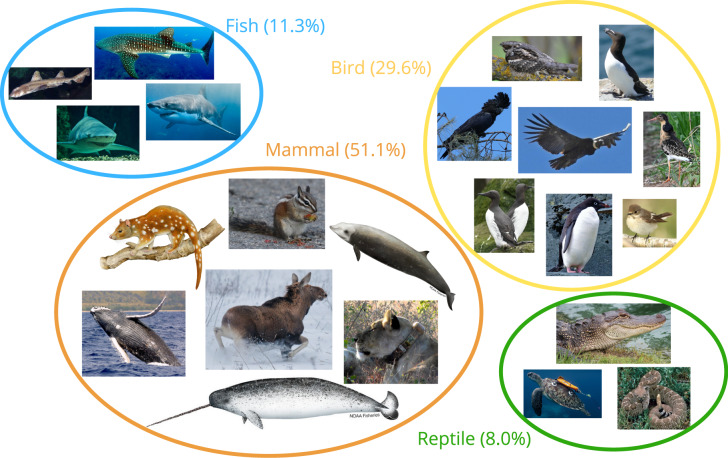
Representation of taxonomic diversity in studies using machine learning and accelerometers for behaviour classification. A diverse range of animals, including both marine and terrestrial species of varying sizes, has been studied. Mammal studies ranged from small animals like quolls and chipmunks to large herbivores (*e.g*., Alaskan moose *Alces alces* gigas), carnivores (*e.g*., African lions *Panthera leo*) and marine mammals (*e.g*., baleen whales, beaked whales and narwhals *Monodon monoceros*). Studies of various terrestrial birds (raptors, passerines, waders and shorebirds), flying seabirds and penguins are included in this review. Fish—especially sharks—but also smaller fish species are also subjects of study. Finally, reptiles represent the smallest proportion of studied species, with most of these studies conducted on marine turtles. Image source credits: Mammals: Northern quoll: Illustration created using Canva. Chipmunk: Henggang Cui, via iNaturalist, CC0 https://www.inaturalist.org/photos/52558374. African lion: Adapted from Wijers et al. (2018), Frontiers in Ecology and Evolution, licensed under CC BY 4.0 (https://creativecommons.org/licenses/by/4.0/). Moose: Лариса Артемьева, via iNaturalist, CC0 https://www.inaturalist.org/photos/610693466. Narwhal: NOAA Fisheries, public domain https://www.fisheries.noaa.gov/species/narwhal. Cuvier’s beaked whale: NOAA Fisheries, public domain https://www.fisheries.noaa.gov/species/cuviers-beaked-whale. Humpback whale: NOAA Fisheries, public domain https://www.fisheries.noaa.gov/species/humpback-whale. Birds: Ruff: Лариса Артемьева, via iNaturalist, CC0 https://www.inaturalist.org/observations/280008890. Red-tailed black cockatoo: Brecht Verstraete, via iNaturalist, CC0 https://www.inaturalist.org/observations/287909838. European pied flycatcher: Tero Linjama, via iNaturalist, CC0 https://www.inaturalist.org/observations/247640304. European nightjar: Лариса Артемьева, via iNaturalist, CC0 https://www.inaturalist.org/observations/78235964. Andean condor: Hugo Hulsberg, via iNaturalist, CC0 https://www.inaturalist.org/observations/253233261. Common guillemot: Лариса Артемьева, via iNaturalist, CC0 https://www.inaturalist.org/observations/176053925. Adélie penguin: Hugo Hulsberg, via iNaturalist, CC0 https://www.inaturalist.org/photos/278396056. Razorbill: Jean-Paul Boerekamps, via iNaturalist, CC0 https://www.inaturalist.org/observations/170678953. Fish: White shark: NOAA Fisheries, public domain https://www.fisheries.noaa.gov/species/white-shark. Horn shark: Ed Bierman, via Wikimedia Commons, public domain https://commons.wikimedia.org/wiki/File:Heterodontus_francisci_SI3.jpg. Lemon shark: Patrick Quinn-Graham, via Wikimedia Commons, licensed under CC BY 2.0 https://commons.wikimedia.org/wiki/File:Negaprion_acutidens_sydney2.jpg. Whale shark: FGBNMS/Eckert, via Wikimedia Commons, public domain https://commons.wikimedia.org/wiki/File:Rhincodon_typus_fgbnms.jpg. Reptiles: Green turtle: Fabien Lefebvre (ACWAA CNRS), used with permission. Western diamondback rattlesnake: Gary Stolz, via Wikimedia Commons, public domain https://commons.wikimedia.org/wiki/File:Western_diamondback_rattlesnake_crotelus_atrox.jpg. American alligator: Mimi Posey, via iNaturalist, CC0 https://www.inaturalist.org/photos/374058532. Created in: Canva Pro, under Canva Pro License.

**Figure 4:**
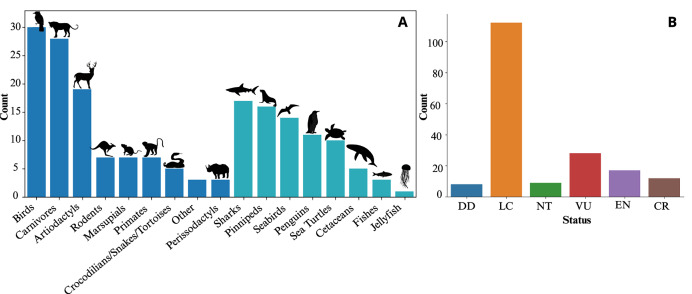
Species represented in acceleration-based behaviour studies. (A) Number of case studies by major animal groups for terrestrial species (dark blue) and marine species (light blue). (B) Bar plot of the international union for conservation of nature (IUCN) red list status of the species studied in this literature review (DD, Data deficient; LC, least concern; NT, near threatened; VU, vulnerable; EN, endangered; CR, critically endangered).

The 125 studies included in our review contained 186 case studies on wild animals. In total, 117 species were represented. More than half of the studies (56.0%) focused on terrestrial species, while 22.4% examined marine species and 18.4% investigated land-breeding marine predators (seabirds and pinnipeds). Four studies included both marine and terrestrial species. Mammals (51.1%) and birds (29.6%) were the most commonly studied taxa, followed by fish and reptiles (Figs. 3 and 4).

Most of the studied species (60.2%) are classified as Least Concern (LC) on the International Union for Conservation of Nature (IUCN) Red List of threatened species, while 35.5% are considered threatened with extinction (Fig. 4). An additional 4.3% are listed as Data Deficient (DD). Some of the most threatened (Critically Endangered; CR) species included in this review are the black rhinoceros (*Diceros bicornis*, Le Roux, Wolhuter & Niesler, 2019), hawksbill sea turtle (*Eretmochelys imbricata*, Jeantet et al., 2018, 2024), northern hairy-nosed wombat (*Lasiorhinus krefftii*, Campbell et al., 2013; Bidder et al., 2014), and Javan slow loris (*Nycticebus javanicus*, Hathaway et al., 2023), amongst others.

#### Geographical range

**Figure 5:**
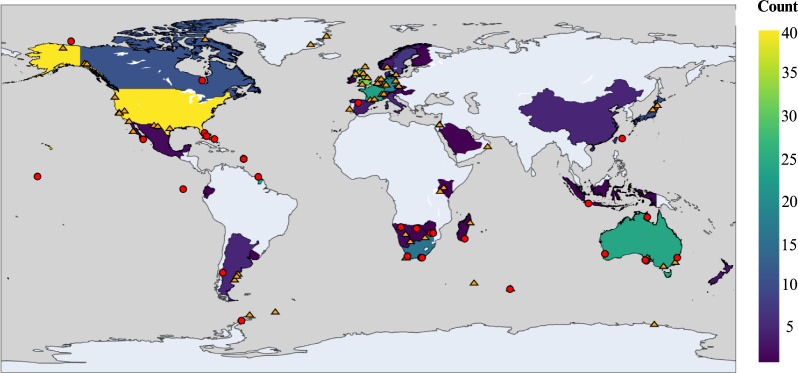
Global distribution of studies using accelerometers and machine learning. Heatmap colours indicate the number of studies per country, based on the affiliations of all authors of the study, while dots mark the locations of wild populations that were studied (red circle, endangered populations; orange triangle, least concern populations) Map source credit: World Bank Group https://datacatalog.worldbank.org/search/dataset/0038272/world-bank-official-boundaries Created in: Canva Pro, under Canva Pro License.

Most research combining accelerometers and machine learning originates from developed countries (*e.g*., the United States, Australia, and European countries), with much of the fieldwork also concentrated in these regions (Fig. 5). In Africa, wild populations studied to date are mainly located in the southern part of the continent. Although it is difficult to evaluate how accessible different populations are based solely on their locations, it is clear that accelerometers and machine learning have been applied to wildlife in several highly remote areas, including Antarctica, Greenland, Alaska, Nunavut (Canada), and Palmyra Atoll in the Pacific Ocean.

#### Studied behaviours

The diversity of studied species can be attributed to the fact that, despite anatomical differences among animals, such as bipeds, tetrapods, apods, and finned species, behaviours can still be distinguished based on body movement and position. As a result, the number of identified behaviours varies across species, depending on how easily they can be differentiated through movement and posture.

For bipeds and quadrupeds, locomotion behaviours such as walking, running, and swimming are generally well detected by machine learning methods. These behaviours produce consistent cyclic patterns due to the rhythmic nature of locomotory movements (Wang et al., 2015; Williams et al., 2015; Giese et al., 2021; Yu & Klaassen, 2021; Yu et al., 2023). The distinct signals are detectable even when accelerometers are deployed *via* neck collars, a common method for tracking terrestrial mammals, in particular. Resting behaviours are also easily distinguished, as they involve minimal movement and result in little to no fluctuation in the accelerometer signal. In contrast, more complex behaviours that lack consistent cyclic patterns and result from a combination of movements and postures, such as feeding, grooming, and preening, are more challenging to identify automatically (Wang et al., 2015; Fehlmann et al., 2017; Pagano et al., 2017; Glass et al., 2020; Giese et al., 2021; Yu et al., 2023). In polar bears (*Ursus maritimus*), for example, feeding on land is characterised by a distinct head-down posture, and this behaviour can be detected with high accuracy. In contrast, polar bear feeding in sea ice habitat was more challenging to identify due to the greater variety of postures and feeding actions involved (Pagano et al., 2017). The difficulty in identifying these complex behaviours may also be due to the limited number of training examples available for the model (Wang et al., 2015; Lush et al., 2016; Fehlmann et al., 2017). To overcome these challenges, some studies have developed binary classifiers that focus exclusively on detecting a specific behaviour of interest, usually feeding or prey capture, while grouping all other behaviours as non-target behaviours (Brisson-Curadeau et al., 2021; Marchand et al., 2021; Sutton et al., 2021; Schoombie et al., 2024).

Studies involving apodal and finned species tend to focus on identifying a smaller number of behaviours. For example only two behaviours were identified for medusa (*Chrysaora fuscescens*, swimming/drifting, Fannjiang et al., 2019), snakes (immobile/mobile, DeSantis et al., 2020), narwhals (buzz/no buzz, Ngô et al., 2021), whale calves (suckling/no suckling, Ratsimbazafindranahaka et al., 2022), Cuvier’s beaked whales (*Ziphius cavirostris*, foraging dive/non-foraging, Sweeney et al., 2022), and whale sharks (*Rhincodon typus*, vertical feeding/non-vertical feeding, Whitehead et al., 2021). For smaller finned species such as sharks (excluding the whale shark), where loggers are attached to the dorsal fin, researchers can differentiate between swimming and resting behaviours in species that exhibit true resting phases (Bouyoucos et al., 2017; Brewster et al., 2018; Karan et al., 2019; Farthing & Yeh, 2022). Some studies have even demonstrated the detection of finer-scale behaviours, such as chewing in Port Jackson sharks (*Heterodontus portusjacksoni*, Kadar et al., 2020) and head-shaking in lemon sharks (*Negaprion brevirostris*, Hounslow et al., 2019). Notably, this review contains no studies on dolphins, although a doctoral thesis on the subject was conducted (Lopes, 2017).

Finally, interaction behaviours are rarely identified using accelerometry, except when a distinct movement or body posture is directly associated with an interaction, such as grooming in chacma baboons (*Papio ursinus*, Fehlmann et al., 2017; Christensen et al., 2023), socio-positive behaviour and fighting in giraffes (*Giraffa camelopardalis*, Brandes, Sicks & Berger, 2021), or nursing in grey seals (*Halichoerus grypus*, Shuert, Pomeroy & Twiss, 2018). Additionally, four studies have investigated the potential for identifying behaviours linked to vocalisations, such as in narwhals (Ngô et al., 2021), in African lions (Wijers et al., 2018, 2021) and in European nightjar (*Caprimulgus europaeus*, Eisenring et al., 2022). The vocalisation of the African lion is detected *via* the distinctive head and neck movements accompanying roaring, whereas in the European nightjar it is identified through body vibrations recorded by accelerometers.

#### Applications

Of the 125 articles reviewed, 60.0% were methodological studies, primarily aimed at developing new algorithms, comparing existing approaches, or adapting established techniques to novel species using data from wild or captive animals (Fig. 2). The remaining 40.0% (50 studies) applied these models to unlabelled datasets from wild populations to estimate and visualise time–activity budgets, that is, the proportion of time allocated to each behaviour. These applications serve three main purposes: demonstrating the effectiveness of methods (16.0%), addressing specific ecological research questions (70.0%), or contributing to conservation efforts (14.0%).

Accelerometer-based behaviour identification is a valuable tool in ecology, enabling researchers to address a wide range of ecological questions (Shamoun-Baranes et al., 2012). A common application is to investigate how environmental factors influence behaviour, often using mixed-effects models to account for individual variation (Brewster et al., 2018; Chimienti et al., 2021; Clermont, Woodward-Gagné & Berteaux, 2021; Hanscom et al., 2023; Lok et al., 2023). For example, Hanscom et al. (2023) investigated the effects of light cycles on kangaroo rat (*Dipodomys merriami*) activity by classifying behaviours such as motionlessness, travel, foraging, and grooming, while Brewster et al. (2018) examined the influence of seasonality and tidal cycles on juvenile lemon shark foraging by identifying swimming, chafing, resting, burst swimming, and feeding behaviours. Combining behavioural data with Global Positioning System (GPS) tracking can further reveal patterns in habitat use, such as spatial patterns of vocalisation in lions (Wijers et al., 2021) or behaviour–environment driven habitat transitions in geese (VonBank et al., 2023). Beyond environmental influences, classifying behaviours such as locomotion, resting, and feeding provides crucial insights into animal energetic strategies. For instance, Ladds et al. (2018) explored compensatory energy strategies in fur seals while Spiegel et al. (2013) studied behavioural responses to food deprivation in griffon vultures (*Gyps fulvus*). Individual variation in time budgets can reflect responses to environmental or social factors (van der Kolk et al., 2021; Minasandra et al., 2023), and when survival data are available, researchers can evaluate the effectiveness of different energetic strategies within a population (Chimienti et al., 2021; van der Kolk et al., 2021). Finally, foraging behaviour, particularly feeding and prey capture, is a key focus in ecological research. Studies on penguins and elephant seals have revealed links between foraging efficiency, prey type, and patch quality (Carroll et al., 2014; Jouma’a et al., 2017; Sutton et al., 2021), and provided valuable insights into predator-prey dynamics (Clermont, Woodward-Gagné & Berteaux, 2021; Schoombie et al., 2024).

In addition to ecological research, accelerometer-based behaviour monitoring is increasingly applied in conservation, where it helps assess the impacts of habitat loss, human activity, and climate change on animal populations, and to inform management strategies. Of the 50 applied studies reviewed, six explicitly used accelerometers and machine learning for conservation purposes. For example, Gooden et al. (2024) studied the energetic costs of cage diving on white sharks (*Carcharodon carcharias*), while Bouyoucos et al. (2017) assessed how longline capture impacts lemon sharks, with the aim to reduce sub-lethal bycatch stress. Lennox et al. (2018) evaluated post-release survival in arapaima (*Arapaima cf. arapaima*) to inform sustainable angling, and Machado-Gaye et al. (2024) identified key foraging areas of Adélie penguins (*Pygoscelis adeliae*) for targeted protection. Yeap et al. (2022) tracked cockatoos to support conservation planning, while Giese et al. (2021) identified cheetah (*Acinonyx jubatus*) kill sites to help mitigate human–carnivore conflict.

### Section 2: Methods used to automatically classify behaviour from accelerometer data

The analysis of the articles identified in this literature review reveals substantial diversity in both the species investigated and the behaviours considered. Despite the specificities of each study, they share a common methodological structure—a general framework for automatic behaviour identification. This framework forms the foundation of most approaches and is composed of a sequence of key steps, each of which can be adapted; many—though not all—have been the subject of targeted optimisation research for specific species or ecological contexts.

The objective of Section 2 is to define this general framework while highlighting the diversity and variation of methods used at each of its steps. In addition, we aim to provide recommendations for each step based on insights from the literature. However, most methods proposed within this general framework, and identified in this literature review, were tested on newly generated datasets and for specific species. Comparative studies spanning multiple datasets and a diversity of species are rare, and validation procedures differ substantially among studies. Moreover, Wilson et al. (2025), in a comprehensive synthesis of validation practices for acceleration-based behavioural classification in both wildlife and domestic animals, demonstrated that many studies applied non-generalising model validation strategies (see ‘Dataset splitting, normalisation, class imbalance, and hyperparameter tuning’ for further details). This lack of standardisation hampers cross-study comparability and the identification of universally optimal approaches. Therefore, the recommendations presented here, which are also informed by our own experience, should be interpreted within this context. In some cases, they require further validation and may vary depending on the species studied and the conditions under which the methods are applied.

#### General methodological framework for acceleration-based automatic behavioural identification using machine learning

**Figure 6:**
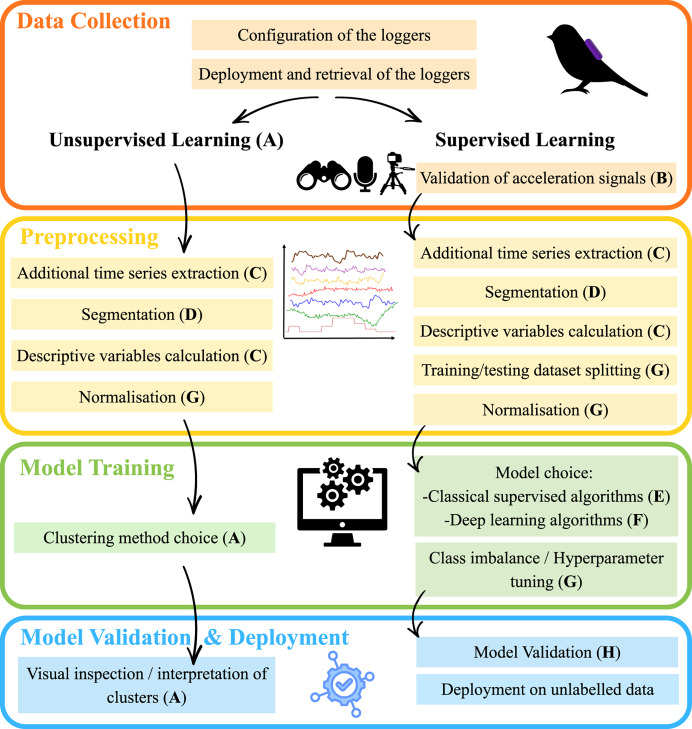
General methodological framework for acceleration-based automatic behavioural identification using machine learning in ecology, highlighting key steps where methodological decisions are required. Capital letters refer to the subsections of Section 2 as follows: A: “Unsupervised vs. supervised learning”; B: “Validation of the acceleration signals”; C: “Additional time-series extraction and computation of descriptive variables”; D: “Segmentation of the acceleration signals”; E: “Classical supervised learning algorithms (excluding deep learning)”; F: “Deep learning algorithms”; G: “Dataset splitting, normalisation, class imbalance, and hyperparameter tuning”; H: “Validation of supervised models”.

Acceleration-based behavioural identification using machine learning is founded on the assumption that each behaviour corresponds to a characteristic movement and posture, thereby generating distinct and recognisable acceleration patterns that can be exploited by machine learning algorithms. This process follows a general methodological framework in which each step is shaped by choices made by the researcher, with some variations depending on whether supervised or unsupervised learning approaches are adopted (Fig. 6).

This framework first involves a set of pre-deployment configurations—such as calibration, sample size, logger placement, and sampling frequency—which are not discussed in detail in this review but are known to substantially influence classification performance (*e.g*., Hounslow et al., 2019; Yu et al., 2023, who investigated the effect of sampling frequency on behaviour identification). Once loggers are deployed and acceleration data are collected, researchers may adopt either supervised or unsupervised learning approaches (see ‘Unsupervised learning *vs* supervised learning’). The key distinction between these approaches is that supervised methods require prior validation of accelerometer signals (see ‘Validation of the acceleration signals’) through the construction of labelled datasets used for model training, whereas unsupervised methods can be applied directly to the collected data to identify behavioural patterns without labelled observations. Signal validation necessitates the simultaneous deployment of loggers and direct observation of animal behaviour. Although supervised learning generally yields reliable and accurate classification, depending on the species and its environment, this step can be logistically and financially challenging, or even infeasible, in which case unsupervised approaches may be more appropriate. Beyond this point, both supervised and unsupervised approaches rely on a shared set of processing steps. These typically include an initial preprocessing step to compute features from the acceleration time series that best capture the relevant information contained in the raw signal (see ‘Additional time series extraction and descriptive variables calculation’). The acceleration time series, together with newly extracted features and data from any additional sensors (*e.g*., gyroscope, magnetometer, pressure sensor), are subsequently segmented into temporal windows (see ‘Segmentation of the acceleration signals’). Each window is assumed to represent a single behaviour that the model will attempt to predict. Within each window, summary statistics are computed to derive descriptive variables that serve as inputs to machine learning algorithms (see ‘Additional time series extraction and descriptive variables calculation’). The next step involves selecting an appropriate algorithm to classify behaviours. For supervised learning, we distinguish between classical supervised algorithms (see ‘Classical supervised learning algorithms (excludes deep learning)’) and deep learning approaches (see ‘Deep learning algorithms’). Classical supervised algorithms do not inherently learn feature representations and therefore rely on hand-crafted descriptive variables (described previously and detailed in ‘Additional time series extraction and descriptive variables calculation’), whereas deep learning algorithms are capable of automatically learning hierarchical feature representations directly from the input data. To evaluate a supervised model’s ability to make predictions on new data, it is essential to partition the labelled dataset, prior to training, into training and testing subsets (see ‘Dataset splitting, normalisation, class imbalance, and hyperparameter tuning’). Model evaluation then depends on the type of learning approach: for supervised models, performance is assessed based on predictive accuracy and generalisation to unseen data (see ‘Validation of the supervised models’), whereas for unsupervised models, clusters are typically examined visually or analytically to determine their correspondence with potential behaviours or behavioural classes. Once a supervised model has been trained and validated, it can be applied to unlabelled data that have been pre-processed in the same way as the labelled dataset.

#### Unsupervised learning *vs* supervised learning

The first application of machine learning to automatically identify behaviour from accelerometer data was published in 2009, using an unsupervised K-means algorithm (Sakamoto et al., 2009). Since then, only 17 of the 125 studies reviewed used unsupervised learning algorithms, while the majority applied supervised learning methods. Nearly all unsupervised studies were conducted on free-ranging animals, with one exception that directly compared unsupervised and supervised methods (Sur et al., 2023). Birds accounted for 11 out of 16 application studies, and the most frequently used techniques were Hidden Markov Models (HMM) and Expectation-Maximisation (EM) (see Box 3 for definitions of unsupervised algorithms).

Box 3 **Time series extracted from acceleration signals**

**Hidden Markov Model (HMM):** A probabilistic model that identifies hidden states, such as animal behaviours, based on sequences of observed data, like the features extracted from accelerometer signals. It assumes that a sequence of observed variables is generated by a set of hidden states, with the observed values depending on the current underlying state.

**Expectation-Maximisation (EM):** An iterative algorithm used to find maximum likelihood estimates in models with latent variables, such as Gaussian Mixture Models (GMMs) for clustering. In a GMM, data points are assumed to be generated from a mixture of Gaussian distributions, each defined by its own set of parameters. The EM algorithm alternates between estimating the probability of cluster assignments (E-step) and updating the model parameters to maximise the likelihood of the observed data (M-step).

**K-means:** A clustering algorithm that partitions data into a specified number of clusters by minimising the sum of squared distances between each point and its assigned cluster centroid. It iteratively updates point-to-cluster assignments and recalculates centroids to improve cluster cohesion.

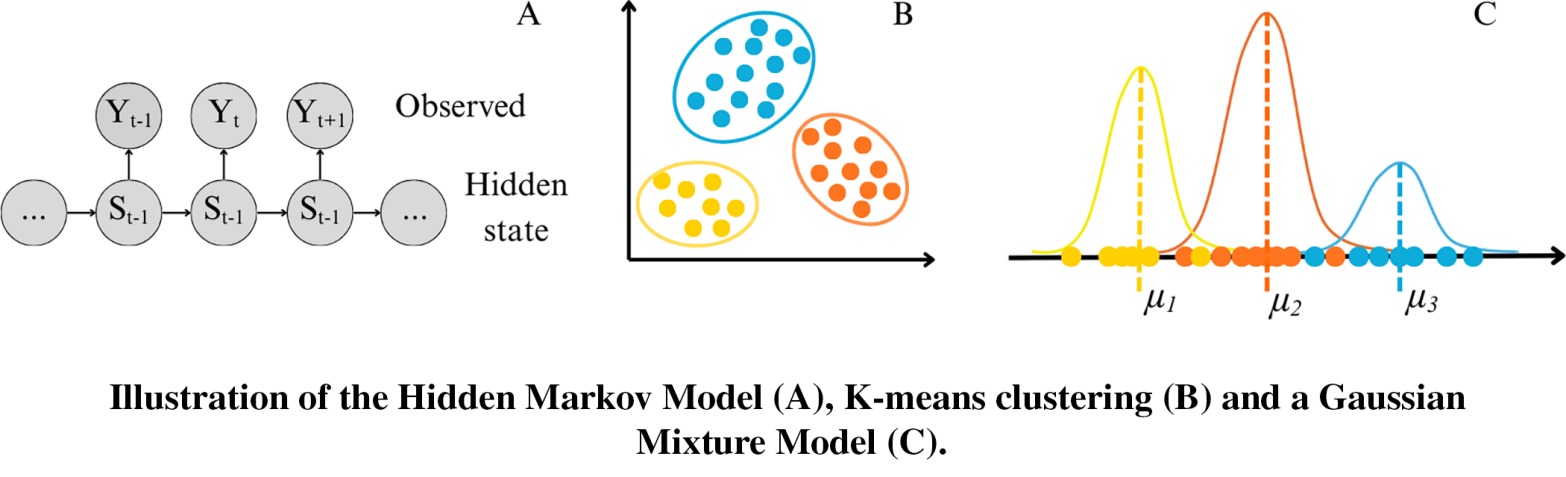

As described in the general framework, unsupervised learning algorithms rely on segmenting the accelerometer data into temporal windows, from which descriptive variables are calculated. Based on the similarity of the calculated variables, the windows are grouped into different clusters, with each cluster assumed to represent a behaviour or activity group. Most algorithms require the number of clusters to be defined in advance. A common strategy is to test different numbers of clusters, visually inspecting the resulting clusters, and selecting the model with a number of clusters that can be ecologically explained (Chimienti et al., 2016; Eisenring et al., 2022; Soldatini et al., 2023). Two to four clusters were identified in more than 50.0% of the unsupervised studies reviewed, sometimes representing specific behaviours such as resting, flying, or foraging (Chimienti et al., 2021; Eisenring et al., 2022; Bullock et al., 2024). When a direct association with a specific behaviour is not possible, clusters are instead linked to general activity levels (Leos-Barajas et al., 2016; Adam et al., 2019; Campera et al., 2019; Papastamatiou et al., 2022). Establishing the ecological significance of each cluster requires visual data inspection combined with in-depth knowledge of the species’ biology (Machado-Gaye et al., 2024).

An important advantage of unsupervised learning over supervised learning is that it does not require labelled data during training, instead it clusters windows of data based on similarity. This can substantially simplify data collection compared to supervised approaches, which require accelerometer signals to be validated through simultaneous behavioural observations—often necessitating extensive fieldwork or the use of on-board cameras to document behaviours (see ‘Validation of the acceleration signals’). The absence of labelling constraints also allows the entire accelerometer dataset to be used for training, whereas supervised learning approaches are often restricted to a smaller labelled subset, given the difficulty of continuously observing free-ranging wildlife.

A key limitation of unsupervised learning is that behaviours assigned to clusters are inferred rather than validated by ground-truth data, making it difficult to assess their accuracy and the precision of model predictions. For example, it may remain unclear how closely an inferred state, such as foraging behaviours, corresponds to prey consumption in marine predators; clusters identified by the algorithms thus represent broad behavioural states or modes, rather than specific behaviours. Sur et al. (2023) highlighted this limitation by testing whether unsupervised algorithms (K-means, EM) could classify predefined behaviours—drinking, flying, sitting, and walking—using a labelled dataset from captive California condors (*Gymnogyps californianus*), and by directly comparing their performance with supervised approaches such as RF, SVM, K-NN, LDA, ANN, and DT (Box 2). The results showed that unsupervised models achieved lower overall accuracy (0.61 for K-means and 0.77 for EM) compared to the best-performing supervised models (*e.g*., RF = 0.91, K-NN = 0.81). Moreover, Cohen’s Kappa scores were markedly lower for unsupervised models (0.02 for K-means and 0.06 for EM) compared to RF (0.83) and K-NN (0.53). The Kappa statistic evaluates the agreement between predicted and true labels, while correcting for agreement that might occur by chance (−1 = complete disagreement, 0 = random classification, 1 = perfect agreement) (Ben-David, 2008). These results highlight the risk of misinterpretation when unsupervised learning is used to classify predefined behaviours, and emphasise the importance of *post hoc* calibration with unsupervised learning (Sur et al., 2023).

Accordingly, where feasible, we recommend prioritising supervised approaches over unsupervised methods, as the latter do not fully resolve uncertainty in behaviour identification and cannot be validated with comparable precision. However, when the validation of accelerometer signals required for supervised learning is not possible, unsupervised methods remain a valuable tool for automatically grouping accelerometer sequences. These groupings can then serve as a basis for subsequent ecological interpretation. In line with the findings of Sur et al. (2023), we recommend interpreting such clusters as broad behavioural states rather than specific behaviours, given that their accuracy cannot be reliably assessed in the absence of a validation phase.

#### Validation of the acceleration signals

The identification of behaviours from accelerometer sequences using supervised algorithms relies on training models with labelled data. Specifically, an important first step involves validating accelerometer signals by associating each sequence with its corresponding behaviour. This process typically requires the simultaneous observation of behaviours during data recording. However, such observation can be particularly challenging in studies of wild species in their natural habitats, where continuous long-term visual monitoring is rarely feasible due to the animals’ mobility and environmental constraints. Consequently, researchers employ a range of methods to validate accelerometer signals, tailored to the specific species and their ecological contexts.

One of the earliest approaches to overcoming the challenges of observing animals in their natural habitats is the use of captive individuals. Nearly half (49.0%) of the studies referencing supervised algorithms validated acceleration signals using captive animals, primarily applied to terrestrial species (73.0% of studies involving captive validation). Captive validation involves equipping animals with accelerometers while observing or video-recording their behaviour in an enclosed environment. Captivity provides the advantage of a controlled environment, enabling extended and uninterrupted data collection. It also facilitates close-range observation, simplifying the deployment of attachment devices and the retrieval of loggers. However, several studies highlight discrepancies in behavioural patterns and acceleration signals between captive and free-ranging conditions, cautioning against the direct application of models trained on captive animals to wild populations (Jeantet et al., 2018; Fannjiang et al., 2019). Captivity does not always accurately reflect natural conditions, as behaviours observed in the wild may be absent in captivity, or vice versa, or be expressed with different postures and movements depending on whether the animal is in the wild or in captivity (Pagano et al., 2017; Jeantet et al., 2018; Rast et al., 2020). Fannjiang et al. (2019) demonstrated through both captive and *in situ* validation that even in organisms displaying simple behaviours, such as swimming and drifting in medusa, the descriptive variables used to classify behaviours differed significantly between laboratory and field data. Similarly, Jeantet et al. (2018) found that dynamic acceleration contributed minimally to behavioural classification in green turtles housed in aquariums, despite being among the most important variables in models trained on free-ranging individuals (Jeantet et al., 2021). Pagano et al. (2017) demonstrated that models trained on captive polar bears performed significantly worse (average F1-score of 49.4%) than those trained on free-ranging individuals (74.3%), highlighting the limitations of using captive-based data for behavioural classification.

Expanding the use of captive animals to validate accelerometry signals, five studies have investigated the feasibility of using captive species different from the species of interest (*e.g*., domestic dogs, *Canis familiaris*, for wolves, *Canis lupus*, brown bears, *Ursus arcto*s, for polar bears, Campbell et al., 2013; Pagano et al., 2017; Ladds et al., 2018; Ferdinandy et al., 2020; Dickinson et al., 2021). Campbell et al. (2013) evaluated the effectiveness of an SVM model trained on domestic dogs in classifying the behaviours across a wide variety of captive wild species. They found that behavioural classification accuracy remained high (>90%) for species with a similar spine length-to-height ratio to that of the surrogate (*e.g*., cheetah), but low for species whose ratio exceeded three times that of the surrogate (*e.g*., Eastern grey kangaroo, *Macropus giganteus*, short-beaked echidna, *Tachyglossus aculeatus*). Dickinson et al. (2021) found that a RF trained on the pygmy goat (*Capra aegagrus hircus*), had low accuracy in predicting behaviours of a phylogenetically similar species, the Alpine ibex (*Capra ibex*). Similarly, Pagano et al. (2017) reported better classification performance for polar bear behaviours when the model was trained on captive polar bears rather than captive brown bears.

Ideally, models should be trained on data that closely match the conditions in which they will be applied. To achieve this, validating acceleration signals using free-ranging animals is the most appropriate approach. Among the 108 studies employing supervised methods, the most commonly used technique for validating signals in free-ranging animals was direct visual observation in the field by researchers and/or video recordings (35.2%). This method typically relies on the use of GPS or Very High Frequency (VHF) to locate tagged animals and observe them in their natural habitat. Observation durations vary widely, ranging from less than 4 h (Shamoun-Baranes et al., 2012; Lush et al., 2016; Hanscom et al., 2023) to over 100 h of behavioural analysis (Grünewälder et al., 2012; Shuert, Pomeroy & Twiss, 2018). Consequently, labelled datasets of comparable size to those obtained in captivity can be generated. However, visual observation of animals in the wild remains logistically demanding, often necessitating substantial resources such as boats, vehicles, or VHF tracking. Moreover, its feasibility depends on the habitat and environmental conditions and may not be possible for all species (Masello et al., 2023; VonBank et al., 2023). Additionally, certain behaviours that are difficult to observe may be underrepresented or entirely absent. Direct visual observation is primarily used for terrestrial animals and is more challenging to apply to marine species. Excluding seabirds, only two studies have validated acceleration signals in marine animals using visual observation of free-ranging individuals (whale sharks and arapaima, Lennox et al., 2018; Whitehead et al., 2021). For terrestrial animals, few studies have integrated data from captive animals with visual observations of wild individuals to enhance behavioural validation (Nathan et al., 2012; Graf et al., 2015).

Since the 2020s, the use of animal-borne cameras has enabled continuous, multi-hour recordings of species for which behavioural validation was previously unfeasible in the wild (*e.g*., green turtles, penguins) or in captivity (*e.g*., whales). Of the 125 studies reviewed, 13 incorporated on-board cameras to validate acceleration signals. This approach has been predominantly applied to marine species, including fishes, sea turtles, sharks, whales, penguins, and flying seabirds (*e.g*., Sutton et al., 2020, 2021; Jeantet et al., 2020, 2021, 2024; Brewster et al., 2021; Ratsimbazafindranahaka et al., 2022; Papastamatiou et al., 2022; Otsuka et al., 2024; Schoombie et al., 2024; Del Caño et al., 2024). To date, only one terrestrial application has been reported, in which Pagano et al. (2017) used a collar-mounted camera to study polar bear behaviour. Since 2021, animal-borne acoustic recorders have also been introduced for both terrestrial (*e.g*., Canada lynx, *Lynx canadensis*, African lions, carrion crows, *Corvus corone*, Wijers et al., 2018, 2021; Studd et al., 2021; Hoffman et al., 2024) and marine species (*e.g*., narwhals, Cuvier’s beaked whales, Ngô et al., 2021; Sweeney et al., 2022). These six studies have documented the integration of acoustic recorders with accelerometers, enabling behavioural identification and validation through the sounds produced by the animals. Beyond studies on penguins and seabirds, where individuals return to their colonies, the successful implementation of on-board cameras has often relied on automated release mechanisms, coupled with VHF tracking and/or Argos beacons for device retrieval. Despite the significant advantages of these technologies, their deployment requires substantial logistical and financial resources, both for equipment acquisition and retrieval in the field. Additionally, sensor malfunctions and tag loss remain challenges, potentially leading to data loss (Pagano et al., 2017; Sutton et al., 2020, 2021; Jeantet et al., 2020; Colson et al., 2024; Schoombie et al., 2024). Analysing the recorded video or acoustic can also be highly labour-intensive and time-consuming.

Accordingly, each validation method reported in the literature has its own advantages and limitations, which may depend on the species studied and the ecological context of application. Ideally, validation should be conducted under conditions that closely match those in which the models will ultimately be used—preferably with free-ranging animals, over extended periods, and using a sufficiently large sample size to minimise the impact of inter-individual variability on model performance (Chimienti et al., 2022). In practice, however, such ideal conditions are often constrained by logistical, financial, and field-related limitations. Researchers must therefore evaluate what is realistically achievable and explicitly consider the limitations of the chosen validation approach when training and applying behavioural classification models.

#### Additional time series extraction and descriptive variables calculation

In ecological studies using machine learning, at least for classical machine learning, behavioural identification begins with the extraction of descriptive variables from raw acceleration signals. This preprocessing step aims to generate variables that are more easily interpretable for behavioural classification and suitable for use as inputs to machine learning algorithms (see Table S1 in the Supplemental Materials for a list of the main descriptive variables used in ecology). These variables, also called handcrafted features, are designed by researchers based on their knowledge of the species, behaviours, and signal characteristics, with the goal of defining metrics that effectively distinguish between behaviours.

Both supervised and unsupervised approaches typically rely on the same descriptive variables, which fall into two main categories: time-domain and frequency-domain features (deep learning can be an exception: see ‘Deep learning algorithms’). Time-domain features, which were frequently used in the studies reviewed, are derived by creating new time series from accelerometric data to capture specific components of the signal. Common new time series that provide information about an animal’s body orientation and movement intensity include static and dynamic acceleration, vectorial dynamic body acceleration (VeDBA), pitch, and roll (Box 4). These time series are segmented into windows, and summary statistics (*e.g*., mean, standard deviation, minimum, and maximum among others, Table S1) are computed for each window to serve as inputs for the algorithms. Frequency-domain features, in contrast, involve transforming the data from the time domain to the frequency domain using fast Fourier transform. This transformation produces an amplitude spectrum from which variables such as spectral energy, dominant frequency, and mean frequency can be calculated (Table S1). These features offer insights into the periodicity of behaviours, such as wingbeat patterns in birds (Sakamoto et al., 2009; Shamoun-Baranes et al., 2012; Yu et al., 2023) or locomotor cycles associated with running and walking in terrestrial animals (Fehlmann et al., 2017; Pagano et al., 2017).

Box 4 **Definition of the main unsupervised machine learning algorithms used in acceleration-based behaviour identification in ecology**

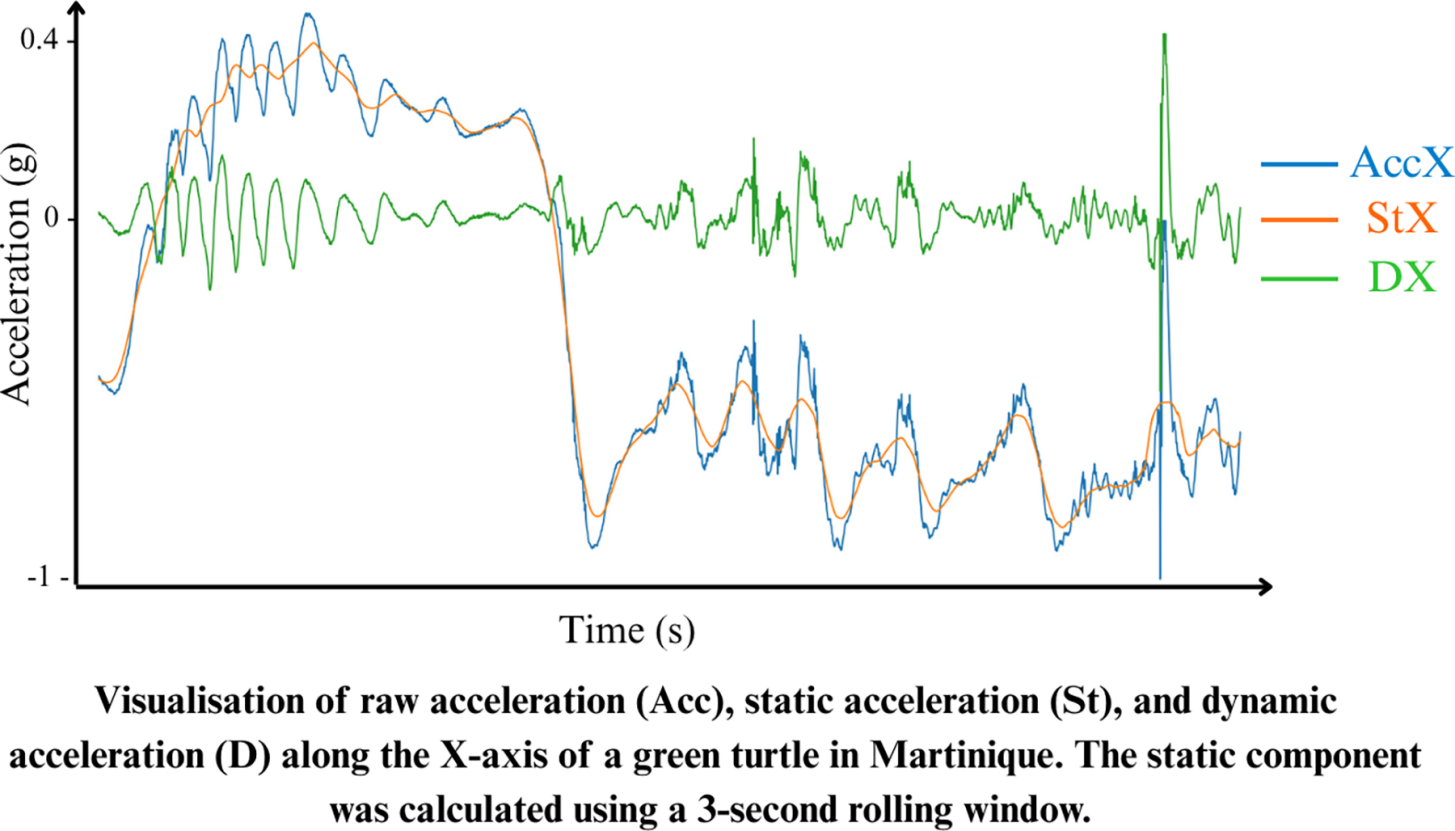

The raw acceleration data (Acc) recorded by the logger are typically composed of two components: static acceleration (St), which reflects the effect of gravity, and dynamic body acceleration (D), which results from the animal’s movement (Shepard et al., 2008b; Wilson, Shepard & Liebsch, 2008; Brown et al., 2013). Static acceleration provides information about the animal’s posture, while dynamic acceleration reflects its motion. Static acceleration is estimated using a moving average (rolling mean), with window size depending on the species (Shepard et al., 2008a). Dynamic acceleration is then obtained by subtracting the static component from the raw acceleration signal:

$AccX = \; StX + \; DX$

$AccY = \; StY + \; DY$

$AccZ = \; StZ + \; DZ$

From the static acceleration, body orientation metrics such as Pitch and Roll can be derived (Wilson, Shepard & Liebsch, 2008). Pitch indicates the angle of the animal’s body relative to the vertical axis, while Roll describes the tilt of the body relative to the horizontal axis:

$Pitch\; = arc\; sin\left( {\displaystyle{{ - StX} \over {\sqrt {St{X^2} + St{Y^2} + St{Z^2}} }}} \right) \times \displaystyle{{180} \over \pi }\;$

$Roll\; = 2\; arc\; tan\left( {\displaystyle{{StZ} \over {StY\; { + ^{}}\sqrt {St{Y^2} + St{Z^2}} }}} \right) \times \displaystyle{{180} \over \pi }\;$

Dynamic acceleration can also be used to estimate the Vectorial Dynamic Body Activity (VeDBA), a proxy for energy expenditure (Qasem et al., 2012; Walker et al., 2015):

$VeDBA = \sqrt {D{X^2} + D{Y^2} + D{Z^2}}$

The selection and number of descriptive variables constitute a critical methodological choice that can substantially influence model accuracy (Le Roux, Wolhuter & Niesler, 2019; Yang et al., 2021). In many studies, the feature set expands rapidly, particularly as modern bio-logging tags increasingly integrate multiple sensors (*e.g*., accelerometers, gyroscopes, and magnetometers). Both time-domain and frequency-domain features can be derived from each data stream, further expanding the set of available descriptors. Across the 125 studies reviewed, the average number of calculated descriptive variables was 38 (standard deviation: ±60), with substantial variation among studies and excluding one clear outlier (de Knegt et al., 2021 with 2,117 variables calculated). Accordingly, several strategies for selecting descriptive variables can be observed across the literature. Some studies compute a large set of variables and subsequently reduce the feature space using dimensionality-reduction techniques, such as Principal Component Analysis (PCA), or feature-selection algorithms. For example, de Knegt et al. (2021) applied feature selection and PCA to reduce an initial set of 2,117 variables (including weather and geographic information system data) to eight principal components. Similarly, others derived between 200 and 399 features from tri-axial accelerometer data and applied feature-selection algorithms to identify the most relevant variables for behavioural classification (Chakravarty et al., 2020; Yang et al., 2021; Aulsebrook, Jacques-Hamilton & Kempenaers, 2024). In contrast, some studies have used all extracted features—ranging from 119 to 252—without reduction (Ladds et al., 2017; Brewster et al., 2018; Ferdinandy et al., 2020; Otsuka et al., 2024). Effective classification has also been achieved with as few as 10 to 20 well-chosen variables, manually selected based on signal analysis and behavioural relevance (Campbell et al., 2013; Marchand et al., 2021; Masello et al., 2023).

Overall, no single methodology can be identified for determining either the type or the number of descriptive variables to compute, as behavioural expression and the associated signal characteristics can vary substantially among species. Although some machine learning algorithms, such as RF and SVM, can handle high-dimensional feature spaces (>200 variables) and are often considered robust to large feature sets (Liaw & Wiener, 2002; Suthaharan, 2016), the inclusion of irrelevant or redundant variables has been shown to degrade model performance. This effect was clearly illustrated by Mansbridge et al. (2018), who examined the performance of RF, KNN, and SVM as the number of features increased from 1 to 44 in classifying grazing behaviour in sheep. SVM performance declined sharply beyond four features, RF performance plateaued around nine features but continued to increase slightly up to 44 features, and KNN showed inconsistent performance across feature sets. Together, these findings highlight that feature selection and dimensionality are important methodological choices based on the chosen algorithm.

One approach that frequently improves classification accuracy is to compute a large set of descriptive variables and subsequently apply feature-selection algorithms to identify those most relevant for machine learning classification (Theng & Bhoyar, 2024). While effective, this approach increases methodological complexity and can be computationally demanding, particularly for methods that rely on repeated model training. Dimensionality-reduction techniques such as PCA provide an alternative means of reducing feature dimensionality but often limit the interpretability of the resulting variables. An alternative strategy, which we recommend, is to identify relevant descriptive variables *a priori* through careful visual inspection and signal analysis, supported by a strong understanding of accelerometer signal validation. Wilson et al. (2018) showed that classification approaches grounded in detailed examination of acceleration signals can outperform non-optimised machine learning methods in both computational efficiency and classification accuracy. This approach allows the number of descriptive variables to be constrained—ideally to fewer than 50—thereby substantially reducing computational costs during model deployment on long-duration datasets, whereas extracting more than 200 features can quickly become prohibitive. However, such manual selection may limit model generalisability, as the chosen variables are often species-specific. Across the literature, descriptive variables related to posture—derived from static acceleration—and power spectral density measures have been identified as among the most informative predictors by RF models in several studies (Fehlmann et al., 2017; Pagano et al., 2017; Shuert, Pomeroy & Twiss, 2018; Fannjiang et al., 2019). Variables related to dynamic acceleration and VeDBA are also important for discriminating behaviours that differ in movement intensity, such as resting *vs* locomotion. More broadly, the literature suggests that effective behavioural classification benefits from a feature set that captures posture, movement intensity, and movement periodicity (Chakravarty et al., 2019; see also the livestock-focused review by Riaboff et al. (2022)).

#### Segmentation of the acceleration signals

The calculation of descriptive variables requires prior segmentation, from which summary statistics can be computed. Consequently, segmentation is an important step, aiming to isolate a single behaviour per segment. Importantly, even when the acceleration sequence is labelled with behaviours, segmentation must be conducted under the same application conditions—that is, without using the labels and without prior knowledge of transitions between behaviours. Segmentation is typically performed with a fixed-size sliding window. The windows may be overlapping (Campbell et al., 2013; Gao et al., 2013) or non-overlapping depending on the study (Aulsebrook, Jacques-Hamilton & Kempenaers, 2024). In addition, determining the appropriate window size poses a significant challenge. Window sizes can range from less than 1 s (Carroll et al., 2014; Chessa et al., 2017) to over 10 s (Bidder et al., 2014; Hammond et al., 2016; Piot et al., 2024), and in some cases, up to 5 min (Grünewälder et al., 2012). Some species exhibit both short- and long-duration behaviours, which are difficult to capture accurately using a single fixed window length. As a result, performance variations across different window sizes have been widely reported (Bom et al., 2014; Ladds et al., 2017, 2018; Resheff et al., 2024), with no consensus on the optimal size. Therefore, selecting a species-appropriate window size is important.

An alternative approach that we recommend is variable time segmentation, where windows are defined based on variations within the data and can differ in size (Bom et al., 2014; Jeantet et al., 2020; Sur et al., 2023; Lok et al., 2023). This method, often driven by change-point algorithms, identifies points where variations in a selected variable exceed a defined threshold, signalling a behavioural transition and the start of a new segment. Implementing this approach requires identifying the most informative variable for distinguishing behaviours and selecting an appropriate threshold. Comparative studies suggest that this method can enhance the classification performance of models compared to fixed-time segmentation (Bom et al., 2014). Other approaches to variable-length time segmentation have been proposed in the HAR literature (Keogh et al., 2001; Li et al., 2025), and their application in ecological contexts would be valuable to explore.

Regardless of whether fixed-size windows or variable-length segments are used, windows containing multiple behaviours are likely to occur (Resheff et al., 2024). Because such mixed-behaviour windows can introduce ambiguity during model training, they are often removed from training and testing datasets. However, this practice does not reflect real-world deployment, where models are applied to unlabelled data and such mixtures cannot be identified *a priori* (Wilson et al., 2025). We therefore recommend retaining these windows during training, for example by introducing a dedicated “transition” class to account explicitly for mixed behaviours (Jeantet et al., 2020).

#### Classical supervised learning algorithms (excludes deep learning)

**Figure 7:**
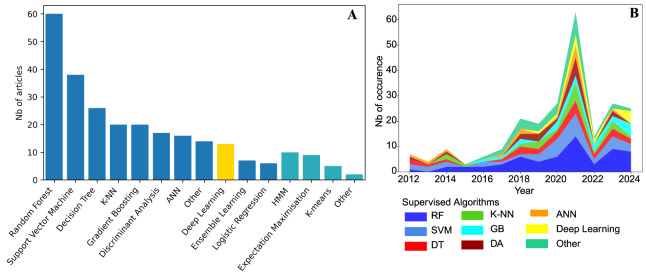
Use of machine learning algorithms for acceleration-based behaviour identification in ecology. (A) Bar plot showing the number of supervised (dark blue) and unsupervised (light blue) algorithms used in acceleration-based behaviour identification in ecology, highlighting the relatively low adoption of supervised deep learning approaches (yellow). (B) Temporal trends in the use of supervised learning algorithms for acceleration-based behaviour identification in ecological studies.

A wide range of supervised algorithms (*n* = 19 over 108 studies using supervised methods) have been applied to identify wildlife behaviours from accelerometer data, using diverse mathematical approaches (see Box 2 for description of the most commonly used ones). Among these, RF was the most frequently applied algorithm (60 out of 125 studies) (Fig. 7A). Nathan et al. (2012) was the first to use RF, in a study that compared the performance of five algorithms (LDA, SVM, DT, RF, and ANN) to automatically identify the behaviour of griffon vultures. This pioneering comparative study demonstrated that RF outperformed the other methods. RF has been the dominant algorithm since 2015 and, despite the introduction of newer methods such as deep learning, it still accounted for 40.0% of studies in 2024 (Fig. 7B).

After RF, SVM and DT were the most frequently used methods in the reviewed studies (Fig. 7A). These methods were predominantly applied during the early adoption of machine learning in ecology to identify behaviours using bio-loggers (2012–2013) (Fig. 7B). While their use has persisted through to 2024, they are more frequently found in comparative studies than in applied studies addressing specific ecological or conservation questions. GB has also become increasingly prominent in ecological studies since 2020. Like RF, GB is an ensemble method based on decision trees; however, the two differ in their training processes and the way they combine the outputs of individual trees (Box 2). Comparative studies have shown that GB performs similarly to RF (Ladds et al., 2017; Jeantet et al., 2020) and can even outperform it for certain species (Ladds et al., 2016; Brewster et al., 2018).

While selecting an appropriate supervised learning algorithm can be challenging, ensemble learning has emerged as a promising alternative in ecological studies since 2017 (Ladds et al., 2017). Used in seven studies, ensemble learning combines predictions from multiple supervised algorithms (*e.g*., RF, SVM, DT, GB, ANN, K-NN), with the final outcome determined by the majority vote of the algorithms included in the ensemble. This approach generally achieves better accuracy and lower variability compared to single algorithms (Ladds et al., 2017; Brewster et al., 2018; Jeantet et al., 2020). Among the seven studies comparing ensemble learning to other methods, five reported superior results using ensemble learning. One disadvantage of ensemble learning is its computational cost, as multiple models must be trained and evaluated to generate predictions. This increases both training and inference time compared with single-model approaches. In addition, ensemble performance depends on the quality and diversity of the constituent models; including weak or poorly tuned learners can reduce efficiency and, in some cases, negatively affect overall performance.

Of the 108 studies using supervised methods, 38 (35.1%) compared the performance of different algorithms for behaviour identification. These comparisons were conducted across different species, and the results indicate that no single algorithm consistently outperforms others, with performance depending on both the species and the algorithms evaluated. Only two studies conducted comparable evaluations of different classical supervised algorithms across multiple species. Yu & Klaassen (2021) assessed six machine learning algorithms (no deep learning) across five different species and found RF, SVM, and GB to deliver similar performance. These three algorithms consistently ranked among the top performers across all datasets, underlining their reliability for behaviour identification in ecological studies. More recently, Hoffman et al. (2024) compared RF, DT and SVM with three different deep learning models across nine datasets of different species, systematically achieving the best results with a deep learning architecture (see ‘Deep learning algorithms’).

Classical supervised machine learning algorithms offer several practical advantages: they are relatively straightforward to implement, their underlying principles are easier to understand than those of deep learning approaches, and—despite sometimes being described as “black-box” methods (Nathan et al., 2012; Brewster et al., 2018)—their decision rules can often be examined to gain insight into behavioural classification. Accordingly, if researchers opt for a classical supervised approach over deep learning, we recommend prioritising RF or GB over the other methods discussed here. These two ensemble methods, both based on decision trees, have consistently demonstrated strong performance across a wide range of species and remain among the most widely used approaches in behavioural classification studies. Compared with SVM, RF is less sensitive to the number of descriptive variables and to the presence of irrelevant features (Mansbridge et al., 2018; Yang et al., 2021; see “Additional time series extraction and descriptive variables calculation”). In addition, the hyperparameters—settings defined by the user—of RF and GB are typically more intuitive and easier to tune than those of SVM. For tree-based methods, these parameters include the number of trees, the number of variables considered at each split, and the minimum node or tree size, whereas SVM performance depends strongly on the choice of kernel and the specification of the decision boundary (Chessa et al., 2017; Ladds et al., 2018). Consequently, for initial applications of machine learning to automatic behavioural identification from accelerometer data, RF and GB provide a robust and accessible entry point. They will perform well for well-characterised behaviours with distinct signal patterns, such as resting and locomotion. However, for more complex behaviours, characterised by less well-defined signals with combinations of multiple postures and movement patterns, the discriminative capacity of RF and GB may be insufficient (Bom et al., 2014; Wang et al., 2015; Pagano et al., 2017; Ladds et al., 2018). In such cases, more advanced approaches—such as ensemble-learning frameworks with carefully selected descriptive variables or deep learning methods—may need to be considered.

#### Deep learning algorithms

Deep learning was first applied to acceleration-based classification of wild animal behaviour in 2019, and by 2024, 13 studies (10.4% of studies in this review) had employed deep learning (Fig. 7), exclusively in the context of supervised learning. Unsupervised deep learning models exist; however, they have not yet been explored in this field. Although overall adoption remains limited, its use has increased in recent years, with approximately one third of studies published in the first half of 2024 applying deep learning (Fig. 7). Notably, 2024 marked a turning point, with several studies introducing novel architectures—such as Transformers, which underpin generative AI models including ChatGPT and Gemini—and deep learning–specific strategies aimed at improving model generalisation (Otsuka et al., 2024; Aulsebrook, Jacques-Hamilton & Kempenaers, 2024; Hoffman et al., 2024; Jeantet et al., 2024).

Among the 13 studies utilising deep learning algorithms, eight studies compared deep learning with traditional algorithms such as RF, SVM, DT, or GB, with deep learning achieving the highest accuracy in six of them (Zhang et al., 2019; Rast et al., 2020; Brewster et al., 2021; Ngô et al., 2021; Otsuka et al., 2024; Hoffman et al., 2024). In ecology, the most frequently used deep learning architecture is the Convolutional Neural Network (61.5%), followed by autoencoder-based architectures such as U-Net or V-Net (38.4%). These architectures rely on convolutional operations, where filters slide over the data to extract hierarchical features through mathematical operations. More recently, sequence-based architectures such as Long Short-Term Memory networks have also been tested on accelerometer time series, as these models explicitly capture temporal dependencies and sequential structure (Otsuka et al., 2024; Aulsebrook, Jacques-Hamilton & Kempenaers, 2024).

A key advantage of deep learning lies in its ability to automatically learn relevant features directly from raw data. Input signals are processed through successive layers of linear and non-linear operations, each learning representations at progressively higher levels of abstraction. During training, millions of parameters are optimised, enabling the model to autonomously extract high-level, discriminative features. As a result, deep learning approaches eliminate the need for manually computing large sets of summary statistics. For example, several comparative studies calculated more than 100 descriptive variables for classical supervised models, while training deep learning models directly on raw accelerometer data (Brewster et al., 2021; Otsuka et al., 2024; Aulsebrook, Jacques-Hamilton & Kempenaers, 2024). In HAR studies, several authors stated that the representations learned by deep learning models are better suited to capturing complex behaviours, whereas hand-crafted features perform well primarily for simple, well-defined activities (*e.g*., walking or running) but are limited in their ability to represent more complex or context-dependent behaviours (Yang et al., 2015; Wang et al., 2019). In addition, advanced architectures such as V-Net and U-Net allow for behaviour prediction at each time point rather than assigning a single behaviour to an entire data window (Jeantet et al., 2021, 2022, 2024; Ngô et al., 2021; Schoombie et al., 2024), reducing the need to isolate behaviours within windows. Sequences cannot be infinitely long, but simple running-window segmentation can be used without isolating individual behaviours (Jeantet et al., 2021). This reduction in preprocessing, combined with the fact that deep learning is particularly well suited to handling large datasets, facilitates the deployment of models on accelerometer data spanning several weeks to several months.

Despite these advantages, deep learning relies on a more complex methodological framework than classical machine learning approaches. Training requires familiarity with concepts such as backpropagation, model architecture design, and optimisation strategies, as well as more advanced programming skills. Deep learning models also involve numerous hyperparameters that strongly influence performance and can be difficult to tune and debug without domain expertise. Moreover, because these models contain large numbers of trainable parameters, they are even more prone to overfitting compared to classical supervised learning algorithms. Overfitting occurs when a model performs well on the training data but poorly on new data, as it becomes excessively tailored to the training data (see ‘Dataset splitting, normalisation, class imbalance, and hyperparameter tuning’). To mitigate overfitting, deep learning typically requires large training datasets, which can be challenging to obtain in ecological studies of free-ranging species. Recent advances in deep learning, highlighted in this literature review, specifically aim to address these limitations through approaches such as data augmentation, transfer learning, and self-supervised learning approaches (Otsuka et al., 2024; Hoffman et al., 2024; Jeantet et al., 2024). Deep learning training is also computationally demanding, often requiring access to high-performance hardware such as graphics processing units (GPUs) to enable efficient parallel computation. The lack of access to, or the high cost of, such infrastructure can represent a significant barrier, although cloud-based platforms such as Google Colaboratory and Kaggle now provide free (albeit restricted) access to GPU resources.

Ultimately, the choice between deep learning and classical supervised algorithms depends on the research question, data availability, and the researcher’s expertise and resources. For many applications—particularly those involving well-characterised behaviours—a carefully selected set of descriptive variables combined with an RF classifier may be sufficient, as reflected by the continued widespread use of RF in the literature. However, for more complex behavioural classification tasks, or when large datasets are available, deep learning offers clear advantages. In cases where researchers lack prior experience with deep learning methods, we strongly encourage interdisciplinary collaborations, particularly with computer scientists, to ensure adapted and effective implementation (see ‘Bridging the gaps in behavioural ecology through interdisciplinary collaboration’).

#### Dataset splitting, normalisation, class imbalance, and hyperparameter tuning

One important risk in machine learning is overfitting, which occurs when a model learns noise or dataset-specific patterns rather than generalisable behavioural signatures. To reliably assess a model’s capacity to generalise, performance must therefore be evaluated on an independent testing dataset that was not used during training. This testing dataset should be labelled, as independent as possible from the training data, and should closely reflect real-world deployment conditions. Multiple strategies exist for partitioning labelled data into training and testing sets, and several studies have shown that classification accuracy can vary substantially depending on the chosen data-partitioning strategy (Ferdinandy et al., 2020; Dickinson et al., 2021; Giese et al., 2021; Aulsebrook, Jacques-Hamilton & Kempenaers, 2024). These have been comprehensively reviewed by Wilson et al. (2025) in their synthesis of validation practices for acceleration-based behavioural classification in wildlife and domestic animals (dogs, cats, and horses). Notably, among the 119 studies they examined, 79.0% did not employ appropriate validation, and thus overfitting could not be reliably detected. This issue most commonly arose due to insufficient independence between training and testing datasets stemming from random data splitting, whereby sequences from the same individuals were included in both datasets. In this literature review, we identified that in 70.4% of studies, data from the same individuals were used for both training and testing. To address this limitation, Wilson et al. (2025) recommend individual-level validation, preferably implemented through cross-validation when dataset size allows. In this approach, entire individuals are iteratively assigned to training and testing roles. A robust method is leave-one-out cross-validation, in which each individual is used once as the test set while the model is trained on all remaining individuals (Hammond et al., 2016; Chakravarty et al., 2020; Kirchner et al., 2023).

Normalisation is an important preprocessing step that is often omitted in ecological studies. It consists of transforming features to a common scale, typically by standardising them to have zero mean and unit variance (Z-score standardisation), to prevent variables with larger numerical ranges from dominating the learning process. Supervised and unsupervised algorithms that rely on distance metrics—such as SVM, K-NN, and K-means—are particularly sensitive to non-normalised data (García, Luengo & Herrera, 2015; Singh & Singh, 2020). Normalisation is also critical for deep learning models, as it improves optimisation stability and facilitates faster convergence toward optimal solutions (Bengio, 2012; Huang, 2022). In contrast, tree-based methods such as DT, RF, and GB rely on feature-threshold splits rather than distances and are therefore largely insensitive to feature scaling, making normalisation unnecessary for these algorithms. One of the most commonly used approaches in machine learning is the Z-score standardisation (Singh & Singh, 2020), applied per feature for classical machine learning models or per sensor axis for raw data in deep learning. Z-score standardisation involves subtracting the mean and dividing by the standard deviation, with both statistics computed exclusively from the training dataset and then applied, without recalculation, to standardise the test data.

Imbalanced datasets are common in acceleration-based behavioural identification in ecology because some behaviours are rare or difficult to observe. This presents a challenge, as many classical machine learning and deep learning algorithms perform poorly under class imbalance. In particular, RF models tend to favour majority classes and often show reduced performance for minority behaviours (Chen, Liaw & Breiman, 2004; He & Garcia, 2009). A common practice in ecological studies is to exclude minority behaviours from the training and testing datasets, but this approach fails to reflect the real-world conditions under which models are ultimately deployed. Retaining all behaviours—by merging similar postures or movements where appropriate, or by grouping infrequent behaviours into an “other” class—does not resolve class imbalance, but it preserves application conditions closer to real-world deployment. In general, modelling approaches that aim to alleviate class imbalance are therefore preferred, as minority behaviours (*e.g*., predator foraging events) are often of primary interest. Although class imbalance is a well-studied problem in other fields (He & Garcia, 2009), it has received limited attention in ecological applications, and no study included in this literature review has explicitly compared different methods to address it.

Three main approaches are commonly used to address class imbalance: oversampling minority classes, undersampling majority classes, and cost-sensitive learning, in which class-dependent weights are assigned to the loss function (*i.e*., the function that quantifies the difference between a model’s predictions and the true labels that the model seeks to minimise during training). Importantly, any modification to class frequencies—through oversampling or undersampling—should be applied exclusively to the training dataset and not to the testing dataset, which is intended to reflect real-world conditions. Aulsebrook, Jacques-Hamilton & Kempenaers (2024) showed that oversampling with Synthetic Minority Over-sampling Technique (SMOTE) generally increased recall (*i.e*., the probability of detecting a behaviour, Box 5) but reduced precision (*i.e*., the proportion of positive predictions that are correct, Box 5). Pagano et al. (2017) tested subsampling dominant behaviours and fully balancing datasets but obtained the best performance using the original imbalanced data. Yang et al. (2021) applied a class-weighted KNN but did not compare it to an unweighted baseline. For deep learning, Otsuka et al. (2024) reported improved performance with data augmentation, while Jeantet et al. (2021) used weighted loss functions without explicit comparison. Despite the availability of numerous imbalance-handling methods—including more than 100 SMOTE variants (Fernandez et al., 2018)—very few have been evaluated in ecological studies. Overall, there is a clear need for systematic assessments of imbalance-handling strategies in acceleration-based behavioural classification, using multiple datasets and rigorous validation to detect overfitting. We encourage researchers to explicitly consider class imbalance and to explore multiple mitigation strategies. For example, weighted RFs—which penalise misclassification of minority classes—or balanced RFs, which sample equally from each class when building trees, can outperform SMOTE and downsampling approaches in some cases (Chen, Liaw & Breiman, 2004). We therefore recommend that researchers consider and evaluate such methods in relation to the specific algorithm and ecological context under study.

Hyperparameter tuning is an important aspect of machine learning, with some studies showing that it can significantly impact model performance (Chessa et al., 2017; Ladds et al., 2018; Yang et al., 2021; Ghate & Hemalatha, 2023). However, these hyperparameters are not always reported in studies. It is also not uncommon for studies to use default hyperparameters provided by machine learning libraries (Rast et al., 2020; Jeantet et al., 2020; Minasandra et al., 2023). We therefore recommend conducting a systematic hyperparameter search (*e.g*., grid search) to identify the most appropriate parameter values. This can be achieved either through manual implementation (Ladds et al., 2017; Ferdinandy et al., 2020; Glass et al., 2020; Painter et al., 2024) or by using automated functions available in many machine learning packages (Sur et al., 2023; Rast et al., 2024). To reduce the computational cost associated with repeated model training, Bayesian optimisation may also be considered for hyperparameter selection (Bergstra, Yamins & Cox, 2013). To avoid overfitting during hyperparameter optimisation, tuning should be performed as part of the training process, prior to final model evaluation. This requires the use of a third, independent subset of data: a validation dataset. Wilson et al. (2025) recommend the use of nested cross-validation at the individual level, with an outer loop defining training and testing datasets, and an inner loop in which individuals from the training set are iteratively assigned to training and validation. This approach requires sufficiently large datasets and Wilson et al. (2025) outline alternative validation strategies when data are limited. We agree with their emphasis on the importance of clearly reporting data-splitting strategies and proportions, validation methods, and performance metrics (see ‘Validation of the supervised models’). Adopting these best practices will promote standardisation in model evaluation and enable more meaningful comparisons across ecological studies.

#### Validation of the supervised models

Once the training, validation, and testing datasets have been properly prepared (see ‘Dataset splitting, normalisation, class imbalance, and hyperparameter tuning’), the model’s predictions on the testing set can be compared to the true labels. This comparison enables the construction of a confusion matrix, from which evaluation metrics are derived (Box 5). A variety of evaluation metrics are available, and selecting appropriate metrics is crucial, as they form the basis for interpreting model performance and determining the most suitable model. This literature review identified more than 13 different evaluation metrics used to validate supervised models, with recall (67.0%), accuracy (66.0%), and precision (59.6%) being the most commonly reported. Recall indicates the model’s ability to detect instances of the target behaviour, Precision reflects the proportion of correct positive predictions, and accuracy represents the overall rate of correct classifications (Rainio, Teuho & Klén, 2024; Miller et al., 2024). These metrics can be computed separately for each behaviour by framing the task as a binary classification, in which the target behaviour is treated as positive and all others as negative, before averaging across behaviours.

Box 5 **Performance metrics for supervised machine learning**

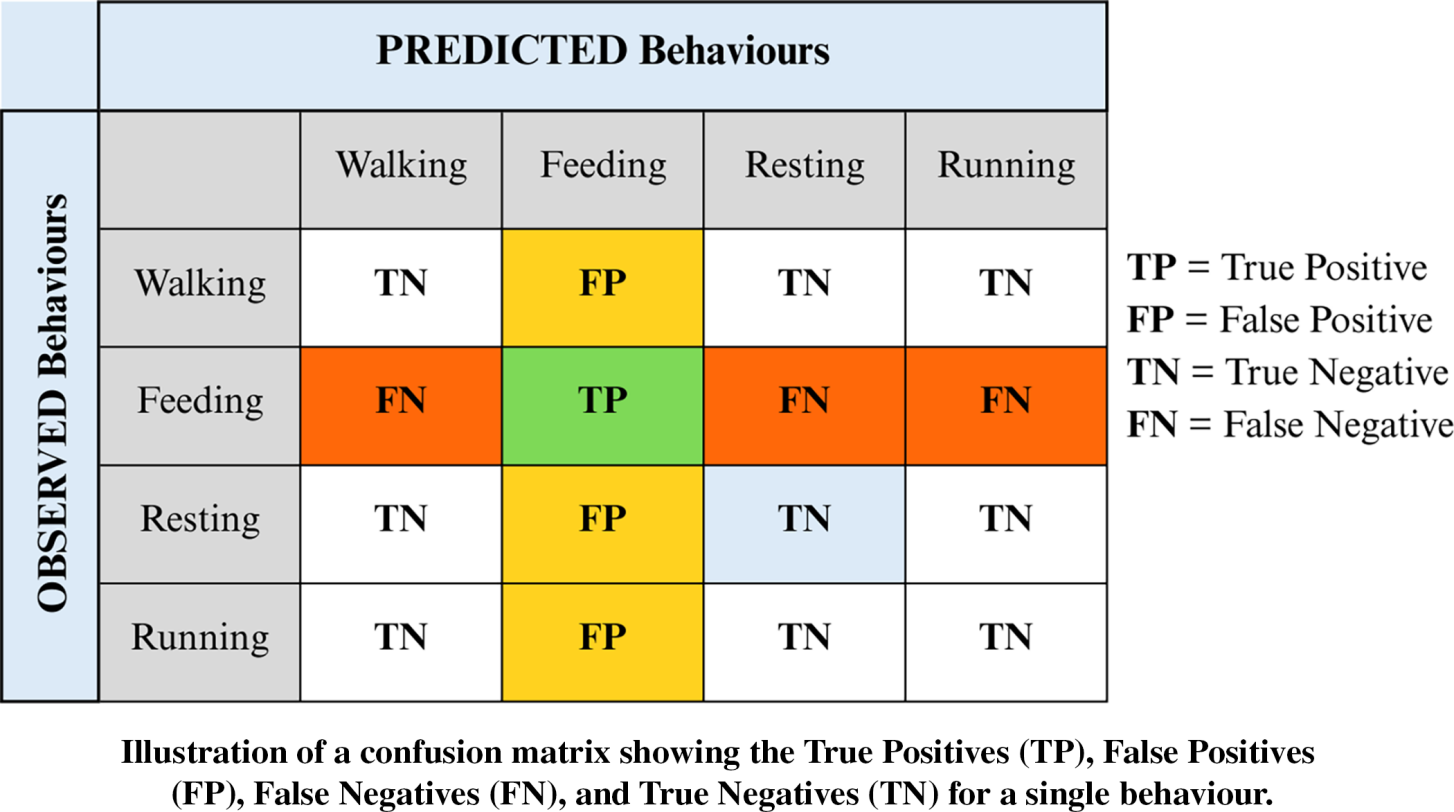

**Accuracy** is the proportion of correctly predicted instances (both positive and negative) out of the total number of instances:

$Accuracy= \displaystyle{{TP+ FP} \over {TP+ FP+ TN+ FN }}$

Accuracy is useful for balanced datasets but may be misleading when classes are imbalanced.

**Recall**, also known as Sensitivity or True Positive Rate, measures the proportion of actual positive instances that were correctly identified by the model:

$Recall = \displaystyle{{TP } \over {TP+ FN}}$

High recall indicates a low false negative rate.

**Precision**, also called Positive Predictive Value, measures the proportion of correctly predicted positive instances out of all predicted positives:

$Precision = \displaystyle{{TP} \over {TP+ FP }}$

High precision indicates a low false positive rate.

The **F1-score** is the harmonic mean of precision and recall. It balances the two metrics and is especially useful when classes are imbalanced:

${F}{1_{{score}}} = 2 \times \displaystyle{{{Precision} \times { Recall}} \over {{Precision } + { Recall }}}$

An F1-score reaches its best value at 1 (perfect precision and recall) and worst at 0.

**Matthews Correlation Coefficient (MCC)** is a balanced measure that takes into account all four confusion matrix categories:

$MCC = \displaystyle{{TP \times TN- FP \times FN} \over {\sqrt {\left( {TP + FP} \right)\left( {TP + FN} \right)\left( {TN + FP} \right)\left( {TN + FN} \right)} }}$

MCC ranges from −1 (inverse prediction) to +1 (perfect prediction), with 0 indicating random prediction.

**The Area Under the Receiver Operating Characteristic Curve (AUC-ROC)** is a performance metric for binary classifiers, obtained by plotting Recall (True Positive Rate) against the False Positive Rate across various decision thresholds and calculating the area under the resulting curve.

$False\; Positive\; Rate = \displaystyle{{FP } \over {FP+ TN }}$

These thresholds correspond to probability cutoffs used to determine whether a prediction is classified as positive for a given behaviour. In multi-class classification, AUC can be extended by computing the area under the ROC curve for all pairs of classes and averaging the results, typically using one-vs-one or one-vs-rest strategies. An AUC value of 1 indicates perfect class separation, while a value of 0.5 reflects performance equivalent to random guessing.

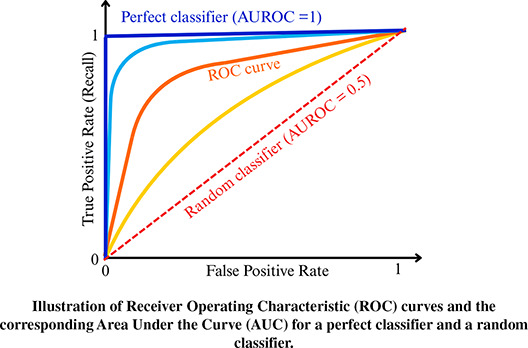

While these metrics are widely used, it’s important to acknowledge their limitations. Notably, accuracy is significantly influenced by imbalanced datasets, often failing to reflect a model’s difficulty to detect rare behaviours (Gu, Zhu & Cai, 2009; Bekkar, Djemaa & Alitouche, 2013; Chicco & Jurman, 2020). For instance, in a dataset with 10 feeding events and 90 resting events, a model that always predicts resting behaviour would achieve 90.0% accuracy, despite failing to detect feeding events entirely. In such cases, the F1-score—defined as the harmonic mean of precision and recall—provides a more informative metric. A high F1-score reflects both strong detection of the target behaviour (*i.e*., high recall, or a large proportion correctly identified) and high precision (*i.e*., few false positives).

Despite its advantages and wide use in other machine learning fields (Chicco & Jurman, 2023), only 24.8% of the reviewed studies reported F1-scores. We therefore recommend increased reporting of F1-scores, particularly given the prevalence of imbalanced datasets in animal behaviour studies. Nonetheless, the F1-score also has limitations, notably it does not account for True Negative values, which can lead to misleading interpretations in highly imbalanced contexts (Chicco & Jurman, 2020). Therefore, in addition to the F1-score, the Matthews Correlation Coefficient (MCC) is also recommended (Chicco & Jurman, 2020; Miller et al., 2024). The MCC measures the correlation between observed and predicted values using all four confusion matrix categories, and it is therefore more informative than both F1-score and accuracy when dealing with imbalanced datasets. However, the MCC remains rarely used in ecological studies, reported in only three studies (Pagano et al., 2017; Nuijten et al., 2020; Brewster et al., 2021), which currently hinders cross-study model comparisons.

Finally, a metric particularly well-suited for comparing algorithms across studies and widely employed in machine learning is the Area Under the Receiver Operating Characteristic (AUROC) curve (Fawcett, 2006; Chicco & Jurman, 2020; Rainio, Teuho & Klén, 2024; Miller et al., 2024). The AUROC metric is generated by plotting recall (*i.e*., the true positive rate) against the false positive rate at various decision boundaries, and calculating the area under the resulting curve. These decision boundaries represent the probability thresholds at which a behaviour is classified as a positive prediction. The AUROC metric can also effectively account for imbalanced class distributions (Hand & Till, 2001) and provides an overall measure of a model’s predictive ability independent of the specific decision boundary, making it highly suitable for comparing models from different studies.

Given these considerations, relying on a single validation metric is inadvisable. Instead, model performance should be assessed using a comprehensive set of metrics, with full consideration of each metric’s limitations and biases (Wilson et al., 2025). It is, for example, considered good practice to report multiple behaviour-specific metrics, such as precision, recall, and F1-score, for each behavioural class. Furthermore, Wilson et al. (2025) recommend reporting the full range of performance variability across validation folds when cross-validation is employed. While MCC appears to be the most informative metric, its limited use in ecology currently restricts meta-analysis. We therefore recommend reporting MCC alongside more commonly used metrics such as F1-score and AUROC.

### Section 3: Identification of gaps and future directions for automatic behavioural identification from accelerometer data using machine learning

#### The need for more general models

This review finds that methodological studies constitute the majority of articles in the field, with 75 of the 125 reviewed studies (60.0%) focusing exclusively on developing methods. The remainder applied acceleration-based behaviour identification to wild populations to test methods or address ecological and conservation questions. Yet, even among these 50 applied studies, only 12 studies employed previously published methods without presenting each step or modifying the overall approach.

This limited reuse of existing approaches likely reflects the complexity of acceleration-based analysis, which typically involves a sequence of steps (Fig. 6, Section 2) that vary according to the species studied and user choices. Consequently, this literature review did not identify a single standardised protocol but rather highlighted the diversity of possible methodological choices and the challenge of converging toward a unified approach.

In addition, meaningful comparisons among existing methods remain challenging, as most approaches are evaluated on different datasets and employ validation procedures that vary substantially across studies, some of which lack sufficient robustness to reliably detect overfitting. As the number of studies in this field continues to increase, it is therefore essential that future work adopts standardised and transparent validation procedures. In particular, new studies should validate their models using the best-practice frameworks outlined in Wilson et al. (2025) and described in ‘Dataset splitting, normalisation, class imbalance, and hyperparameter tuning’ and ‘Validation of the supervised models’ of this review, to facilitate reproducibility and meta-analysis.

From a methodological perspective there are opportunities to develop methods that better generalise across a range of species. The current diversity of species-specific methods may partly result from the historical absence of standardised benchmarks against which new approaches could be systematically tested and compared. This gap has only recently been addressed, with the first benchmarking initiatives emerging in 2024. Notably, Hoffman et al. (2024) introduced the Bio-logger Ethogram Benchmark (BEBE), an open-access resource of nine accelerometer datasets labelled with behaviours, from diverse taxa, including snakes, sea turtles, seals, whales, bears, birds and humans. As evaluating a new method on a novel species or dataset is inherently challenging, this benchmark provides practitioners with both a dataset for developing or refining methods and a framework to systematically evaluate the generalisability of new approaches across multiple species. Developing generalisable rather than species-specific accelerometer-based behavioural identification methods represents a key step toward facilitating their application and promoting their broader adoption within the research community.

#### Advancing toward real-time monitoring

An important next step in bio-logging is the development of on-board data processing capabilities that enable behavioural identification directly on the device (Watanabe & Papastamatiou, 2023). These advancements are already well-established in HAR and livestock monitoring (Zeng et al., 2014; Gutierrez-Galan et al., 2018; De Leonardis et al., 2018; Qi, Su & Aliverti, 2020), and their wider adoption in ecology could offer valuable benefits. For instance, on-board behaviour classification would allow the remote transmission of predicted behaviours and enable real-time monitoring, eliminating the need for device retrieval and animal recapture; an important advantage for species that are difficult to re-encounter, such as marine animals or those undertaking long-distance migrations. This approach opens new avenues for investigating behavioural processes that have so far remained inaccessible and holds considerable potential for conservation, particularly in situations requiring immediate action, such as real-time snaring or poaching detection (Wall et al., 2014; de Knegt et al., 2021).

Data transmission is currently possible *via* Global System for Mobile Communications (GSM), Argos and Iridium Satellite Communications, already commonly used for transmitting GPS locations and dive depth data in marine species (Carter et al., 2016; Chung, Lee & Lee, 2021), as well as short-range networks such as VHF, Bluetooth, Wi-Fi, or LoRa (Allan et al., 2018; Parlin et al., 2025). However, these systems can demand close proximity, have high energy requirements, or are limited by insufficient bandwidth to transmit high-frequency accelerometer data (Chung, Lee & Lee, 2021). This is a key constraint, as machine learning approaches for behaviour identification typically rely on sequences recorded at high sampling rates (*e.g*., 1 Hz to 100 Hz, with an average of 45 Hz in this review). Important progress has been made in marine systems, where researchers have successfully transmitted accelerometry summaries *via* satellite from loggers deployed on seals, and extracted behavioural information from these summaries (Cox et al., 2017; Heerah et al., 2019). By computing the classification on-board and transmitting only the predicted behaviours represents a critical stepping stone to enable real-time ecological monitoring.

On-board automatic behaviour identification presents a new challenge, as the machine learning algorithms must be executed on the logger, with far lower computational power and memory capacity than a standard computer. Moreover, since these loggers rely on battery power, it is essential that these processes are energy-efficient to allow for sufficiently long deployment periods. While research on on-board processing already exists in ecology, it remains limited. We identified only two studies focused on the development of methods for on-board classification, and four application-based studies demonstrating real-time monitoring with on-board behaviour identification. Specifically, Le Roux, Wolhuter & Niesler (2019) and Yu & Klaassen (2021) investigated the energy consumption and processing time of various algorithms on microprocessors, as well as the computational demands of descriptive variable extraction. With an application of on-board classification, Korpela et al. (2020) and Tanigaki et al. (2024) deployed DT algorithms on loggers equipped with both accelerometers and cameras to automatically detect target behaviours of seabirds from accelerometer data and activate video recording only when those behaviours occurred. In Yu et al. (2022a, 2022b) the authors tested a newly developed solar-powered accelerometer logger, weighing 25 grams, capable of automatically classifying the behaviours of Pacific black ducks (*Anas superciliosa*) using an on-board GB algorithm. The logger was able to transmit them *via* mobile phone networks or to store behavioural summaries for up to 7 months. These studies represent an important step forward and those advancements could be extended to other wildlife species. Further research is needed to fully explore the potential of on-board preprocessing for practical applications in real-time monitoring. Deep learning, with its ability to operate without extensive preprocessing, emerges as a promising approach. However, to ensure feasibility in field conditions, the development of lightweight, energy-efficient models will be essential.

#### Bridging the gaps in behavioural ecology through interdisciplinary collaboration

Overall, the field of automatic behaviour identification from accelerometer data in ecology lags significantly behind developments in human and livestock research. For instance, deep learning was first applied to ecological studies only in 2019, whereas its use in HAR dates back to 2011 in an unsupervised context and to 2014 in supervised applications (Plötz, Hammerla & Olivier, 2011; Zeng et al., 2014), and as early as 2012 for livestock monitoring (Nadimi et al., 2012). To date, several literature reviews already exist on the use of deep learning for human activity recognition and livestock monitoring (Wang et al., 2019; Zhang et al., 2022; Mao et al., 2023), while the present work is the first to review the general methodological approaches used in ecology. Deep learning techniques developed for human activity recognition are already considerably more advanced and diverse than those currently applied in ecology (Zhang et al., 2022; Otsuka et al., 2024). This delay in acceleration-based behaviour identification in ecology can be attributed, in part, to the inherent challenges of studying wild species, in some cases endangered species. Deploying and retrieving animal-attached loggers in such contexts is often logistically complex, time-consuming, requires highly specialised skills, and can be costly. As a result, ecological datasets are more difficult to obtain and are typically smaller than those used in human or livestock research, posing greater challenges for method development. These constraints can be further exacerbated when studying small-bodied wild species, which require lightweight, miniaturised loggers that remain limited by battery capacity and recording duration (Hammond et al., 2016; Hanscom et al., 2023). Similar challenges arise for cryptic species, where limited baseline ecological knowledge and low detectability further constrain data collection and study design. Additionally, unlike ecology, both HAR and livestock monitoring are driven by industrial applications, which provide substantially greater funding and resources to support methodological development and innovation (Lee & Kim, 2022; Zhang et al., 2022).

In practice, ecologists rarely draw on advances from HAR or livestock research, and few studies in ecology reference the work conducted in these fields (see citation analysis and Fig. S1 in the Supplemental Materials). Nonetheless, accelerometer-based behaviour identification in humans or livestock relies on the same methodological framework identified in this review and faces similar challenges in selecting appropriate methods at each step (Riaboff et al., 2022). Therefore, there is considerable potential for ecology to benefit from advances in other fields where methodological developments can be readily adapted and reused. For example, the first study to apply a deep learning U-Net architecture to narwhals was inspired by its previous use on multidimensional time series in HAR (Perslev et al., 2019; Ngô et al., 2021). Similarly, Aulsebrook, Jacques-Hamilton & Kempenaers (2024) adapted a deep learning model originally developed for HAR (Bock et al., 2021) to identify the behaviour of ruffs (*Calidris pugnax*). Transfer learning is already widely used in human activity recognition (HAR) and has been explored in livestock research (Hernandez et al., 2020; Kleanthous et al., 2022; Bloch et al., 2023) before being implemented in ecological studies (Jeantet et al., 2024). However, such examples of cross-disciplinary adaptation of methods from HAR and livestock monitoring remain limited. While direct one-to-one transfers of methods may not always be feasible or appropriate, we encourage greater engagement with these fields to explore how comparable methodological challenges have been addressed.

Wildlife acceleration-based behaviour identification lies at the crossroads of ecology, engineering, and computer science, where progress has historically depended on technological innovation (Watanabe & Papastamatiou, 2023). Because no single researcher can be an expert in all these domains, interdisciplinary collaboration is essential for rapid progress. Supervised machine learning models depend on robust ground-truth data, which can only be obtained through ecologists’ detailed knowledge of the study species and its behavioural repertoire. Conversely, computer scientists contribute essential expertise in model design, signal processing, and algorithmic optimisation. Progress in sensor technology also plays a critical enabling role in this interdisciplinary framework. Continued advances in miniaturisation are necessary to reduce logger size and mass preserving sufficient battery life, memory capacity, and recording performance. Partnerships, at the intersection of ecology, technology, and artificial intelligence, are increasingly encouraged in related fields, with repeated calls for closer collaboration in recent years (Carey et al., 2019; Christin, Hervet & Lecomte, 2019; Tuia et al., 2022; Watanabe & Papastamatiou, 2023; Chimienti et al., 2025). The findings of this review reinforce these appeals, underscoring the continuing need to strengthen collaboration among ecologists, engineers, and computer scientists to fully harness deep learning, advance on-board data processing, and enable real-time wildlife monitoring. Such cross-disciplinary efforts will be critical to drive future progress and translate technological advances into meaningful applications for wildlife research and conservation.

#### Limits of acceleration-based behaviour identification in ecology

Behavioural identification using accelerometers and machine learning is a powerful tool for studying wildlife, yet it remains subject to several limitations. One major challenge is the identification of rare, short, or complex behaviours. Supervised machine learning models rely on robust ground-truth data, but many ecologically important behaviours are difficult or impossible to observe in the field. Consequently, these behaviours are often under-represented or absent from training datasets, leading to models that perform well for common and easily observable behaviours but poorly for rare events (Bom et al., 2014; Wang et al., 2015; Aulsebrook, Jacques-Hamilton & Kempenaers, 2024). Although specialised approaches have been proposed to detect rare behaviours, such methods remain limited and are typically context- or species-specific (Chakravarty et al., 2020; Tanigaki et al., 2024). In addition to rare behaviours, unknown behaviours—absent from the training data but encountered during model deployment—are poorly handled by supervised algorithms, which inevitably misclassify them into known classes. In the ecological literature, only one study initially addressed this issue by proposing a framework to account for behaviours unknown to a model trained on captive wolverines (*Gulo gulo*) and applied to wild individuals (Glass et al., 2020). More recently, two additional studies have explicitly considered unknown behaviours or uncertainty during inference (Wilson et al., 2026; Agarwal et al., 2026).

Acceleration-based methods are also constrained in their ability to capture behavioural interactions. Because accelerometers record movement and posture only, they provide no direct information on social context, environmental interactions, or behavioural motivation. To investigate behavioural interactions, complementary bio-logging or bio-telemetry approaches may be employed, often requiring the simultaneous deployment of devices on multiple individuals (Watanabe & Papastamatiou, 2023). Nevertheless, direct visual observation in the field may remain the only way to fully characterise such interactions.

Finally, acceleration-based behaviour identification is inherently species-specific. Logger configuration, attachment methods, deployment strategies, and signal interpretation all require detailed knowledge of morphology, ecology, and natural history of the studied species. This reliance on detailed species knowledge can contribute to a taxonomic bias toward well-studied and accessible species (Figs. 3 and 4). Moreover, the potential impacts of accelerometer deployment on animal behaviour, physiology, and welfare must be carefully considered. These ethical concerns are widely discussed in the bio-logging literature (Ropert-Coudert & Wilson, 2005; Fehlmann & King, 2016; Watanabe & Papastamatiou, 2023), and should remain central to the design and interpretation of any study using accelerometers to investigate the behaviour of wild animals.

#### Behavioural data contributing to conservation efforts

The research field of Conservation Behaviour emerged around 30 years ago (Berger-Tal et al., 2011; Amorim, 2025), and today, behavioural data are an essential part of wildlife conservation and management strategies (Riley et al., 2006; Rode, Farley & Robbins, 2006; Berger, 2007; Buchholz, 2007; Cooke et al., 2023). Acceleration-based behavioural identification represents a relatively new component of Conservation Behaviour, but is particularly important due to its ability to monitor animal behaviour in their natural habitats over extended periods. Numerous reviews in conservation science have emphasised the importance of bio-logging technologies to enhance our understanding of endangered species (Cooke, 2008; Wilson et al., 2015; Ellis-Soto et al., 2025). This need is especially pronounced for marine species, as bio-logging has transformed the study of these often highly mobile and deep-diving animals, which are otherwise challenging to access and monitor in their natural habitats (Watanabe & Papastamatiou, 2023; Chimienti et al., 2025). This literature review revealed that 35.7% of the species studied are listed on the IUCN Red List (Fig. 4B). These findings underscore the strong potential of the method for studying endangered species, even within wild populations inhabiting remote areas (Fig. 5), and highlight the importance of continuing to develop its use for conservation purposes. However, our analysis also showed that very few studies have been conducted in Central Africa, South America, and Asia (Fig. 5), despite these regions harboring the highest species richness (Hughes et al., 2021). Since acceleration-based behaviour identification requires significant logistical and financial resources, it is currently conducted predominantly in developed nations. Expanding these conservation efforts to less developed regions through international collaborations and knowledge transfer is therefore essential.

## Supplemental Information

10.7717/peerj.21069/supp-1 Supplemental Information 1 Supplementary materials.

10.7717/peerj.21069/supp-2 Supplemental Information 2 Lists of both the livestock and wildlife studies using machine learning to automatically identify behaviours from accelerometer data. The complete list of articles studied in this literature review along with the information extracted from each study (only for the studies on wildlife).

## Additional Information and Declarations

### Data Availability

The following information was supplied regarding data availability:

This is a literature review.
