# Supplementary material for: Towards automated behaviour monitoring in wildlife: a review of machine learning approaches using accelerometer data
Source: PeerJ. 2026 May 11;14:e21069. doi: 10.7717/peerj.21069 (PMC13175066; doi:10.7717/peerj.21069)
Supplement: Supplemental Information 1 [file peerj-14-21069-s001.docx]

**Supplementary Materials**

**Table S1:** **Summary of descriptive variables derived from accelerometer data for behavioural classification**. Where x represents the acceleration signal within a given time window; N is the number of samples in the window; FFT(x) is the Fast Fourier Transform of the signal; PSD(x) is the power spectral density derived from the Fourier transform; f denotes frequency; fdom is the dominant frequency (i.e. the frequency at which PSD is maximal); and P(f) represents the normalised power at frequency f (used to compute spectral entropy).

| **Variable** | **Domain** | **Formula** | **Definition** |
| --- | --- | --- | --- |
| Mean | Time | $\mu=\frac{1}{N}\sum_{i=1}^{N} x_{i}$ | Average acceleration within a window |
| Standard deviation | Time | $\sigma=\sqrt{\frac{1}{N}\sum_{i=1}^{N} {(x_{i}-\mu)}^{2}}$ | Measures signal variability around the mean within a window. |
| Variance | Time | $\sigma^{2}$ | Squared dispersion of the signal; another measure of variability. |
| Median | Time | med(x) | Central value of the signal distribution; robust to outliers. |
| First and third quartiles | Time | Q_1_​,Q_3_​ | Values delimiting the lower and upper 25% of the signal distribution. |
| Interquartile range | Time | IQR=Q_3_​−Q_1_​ | Robust measure of signal spread. |
| Maximum | Time | max(x) | Highest recorded acceleration value within a window. |
| Minimum | Time | min(x) | Lowest recorded acceleration value within a window. |
| Range | Time | max(x) – min(x) | Total amplitude span of the signal within a window. |
| Skewness | Time | $\frac{1}{N}\sum_{i=1}^{N} {(\frac{x_{i-\mu}}{\sigma})}^{3}$ | Measures asymmetry of the signal distribution around the mean. |
| Kurtosis | Time | $\frac{1}{N}\sum_{i=1}^{N} {(\frac{x_{i-\mu}}{\sigma})}^{4}$ | Quantifies the tailedness of the distribution relative to normality. |
| Spectral energy | Frequency | $E=\frac{1}{N}\sum_{i=1}^{N} {PSD\left( f_{i} \right)}^{2}$ | Total signal power distributed across frequencies; reflects overall movement intensity in the frequency domain. |
| Spectral entropy | Frequency | $H=\sum_{i=1}^{N} P\left( f_{i} \right)logP(f_{i})$ | Measures spectral complexity or irregularity of movement patterns. |
| Dominant power spectrum | Frequency | max(PSD(f)) | Maximum power value in the power spectral density; indicates the strongest oscillatory component. |
| Dominant frequency | Frequency | f_dom_​=argmax(PSD(f)) | Frequency at which the PSD reaches its maximum; represents the main periodic movement component. |
| Amplitude of dominant frequency | Frequency | ∣X(f_dom_​)∣ | Magnitude of the signal at the dominant frequency. |

Citation Analysis

We performed the citation analysis using VOSviewer software version 1.6.2 (van Eck & Waltman, 2010), based on all articles retrieved from the Web of Science database on July 7, 2025, using the search query: (acceler* OR sensor OR biologger OR bio-logger OR gyroscope) AND ("machine learning" OR "supervised learning" OR "unsupervised learning” OR "deep learning" OR "automatic identification" OR "neural network") AND (animal OR bird OR cetacean OR mammal) AND behavio*

From the initial set of 350 studies, we retained only those cited more than 5 times, resulting in a final set of 253 studies used to construct the citation network. The studies with no link were not represented in the plot. Citation analysis illustrates the relationships between articles by identifying which papers cite others. The analysis identified 17 clusters; articles on acceleration-based behavioural identification applied to wildlife are clustered on the right, while research on livestock is grouped on the left. The network shows few connections between the two fields, with ecology papers predominantly citing other ecology studies.


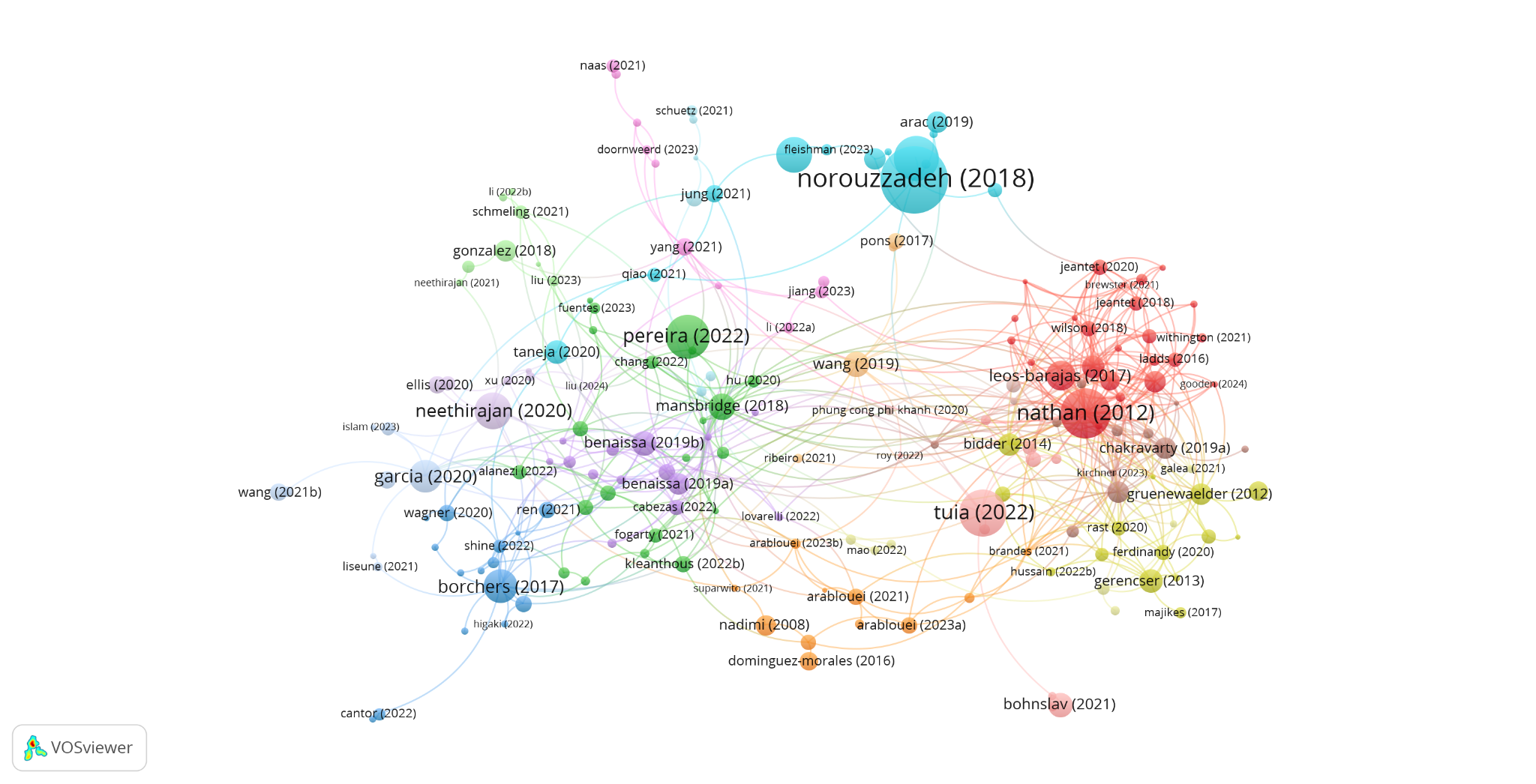


**Figure S1: Citation analysis of articles retrieved from Web of Science, conducted on VOSviewer and revealing 17 clusters.** Clusters are formed based on the number of links between articles, with the size of each node proportional to its number of connections. A link represents either that an article cites, or is cited by, the connected article.

**References:** van Eck NJ, Waltman L. 2010. Software survey: VOSviewer, a computer program for bibliometric mapping. *Scientometrics* 84:523–538. DOI: 10.1007/s11192-009-0146-3.
